# The architecture of the human default mode network explored through cytoarchitecture, wiring and signal flow

**DOI:** 10.1038/s41593-024-01868-0

**Published:** 2025-01-28

**Authors:** Casey Paquola, Margaret Garber, Stefan Frässle, Jessica Royer, Yigu Zhou, Shahin Tavakol, Raul Rodriguez-Cruces, Donna Gift Cabalo, Sofie Valk, Simon B. Eickhoff, Daniel S. Margulies, Alan Evans, Katrin Amunts, Elizabeth Jefferies, Jonathan Smallwood, Boris C. Bernhardt

**Affiliations:** 1 McConnell Brain Imaging Centre, Montreal Neurological Institute, McGill University, Montréal, Quebec Canada; 2 Institute for Neuroscience and Medicine (INM-7), Forschungszentrum Jülich, Jülich, Germany; 3 Translational Neuromodeling Unit (TNU), University of Zurich and ETH Zurich, Zurich, Switzerland; 4 Max Planck Institute for Cognitive and Brain Sciences, Leipzig, Germany; 5 Institute for Systems Neuroscience, Heinrich Heine Universistät Dusseldorf, Dusseldorf, Germany; 6 Integrative Neuroscience & Cognition Center (INCC – UMR 8002), University of Paris, Centre national de la recherche scientifique (CNRS), Paris, France; 7 Institute for Neuroscience and Medicine (INM-1), Forschungszentrum Jülich, Jülich, Germany; 8 Department of Psychology, University of York, York, UK; 9 Department of Psychology, Queen’s University, Kingston, Ontario Canada

**Keywords:** Cognitive neuroscience, Computational neuroscience

## Abstract

The default mode network (DMN) is implicated in many aspects of complex thought and behavior. Here, we leverage postmortem histology and in vivo neuroimaging to characterize the anatomy of the DMN to better understand its role in information processing and cortical communication. Our results show that the DMN is cytoarchitecturally heterogenous, containing cytoarchitectural types that are variably specialized for unimodal, heteromodal and memory-related processing. Studying diffusion-based structural connectivity in combination with cytoarchitecture, we found the DMN contains regions receptive to input from sensory cortex and a core that is relatively insulated from environmental input. Finally, analysis of signal flow with effective connectivity models showed that the DMN is unique amongst cortical networks in balancing its output across the levels of sensory hierarchies. Together, our study establishes an anatomical foundation from which accounts of the broad role the DMN plays in human brain function and cognition can be developed.

## Abstract (web summary)

The default mode network (DMN) is implicated in cognition and behavior. Here, the authors show that the DMN is cytoarchitecturally heterogeneous, it contains regions receptive to input from the sensory cortex and a core relatively insulated from environmental input, and it uniquely balances its output across sensory hierarchies.

## Main

The default mode network (DMN) is a distributed set of brain regions in the frontal, temporal and parietal lobes with strongly correlated fluctuations^1^. It is among the most influential, yet challenging, discoveries of modern neuroscience. Theories on the role of the DMN initially focused on internally oriented cognition and its antagonism with task-positive networks^2,3^, but increasing evidence shows DMN activity is related to the content of external stimuli^4,5^ and externally oriented task demands^6–8^. Additionally, DMN subregions can cofluctuate with regions of task-positive networks^9,10^. Thus, the DMN poses a conceptual challenge: how can a neural system be involved in so many different states, particularly as many seem antagonistic, such as perceptually driven decision-making^11^ and perceptually decoupled cognition^12–14^?

Recent perspectives have argued that resolving the role of the DMN in cognition depends on understanding its anatomy^3,15,16^ because neuroanatomical insights can narrow the search space for conceivable theoretical accounts of its function. Although the DMN is typically defined on functional grounds (that is, strong resting-state functional connectivity and relatively lower activity during externally oriented tasks), its subregions are also connected by long-range tracts^17,18^ and each subregion is maximally distant from primary sensory and motor areas^19^. This topography may allow activity in the DMN to be decoupled from perception of the here and now^15^, as neural signals are transformed incrementally across cortical areas from those capturing details of sensory input toward more abstract features of the environment^20,21^. These observations suggest neural activity in the DMN has the potential to be both distinct from sensory input, while also incorporating abstract representations of the external world. This could explain the network’s involvement across diverse contexts^15^. Although this topographical perspective, in principle, accounts for its broad involvement in human cognition, we lack a detailed explanation of how the neural circuitry in the DMN enables this hypothesized role.

Given the highly distributed nature of the subregions of the DMN, it is likely to be heterogeneous in terms of its microarchitecture; however, the specific nature of this heterogeneity remains unknown. On the one hand, it is conceivable that regional differences in the DMN are most pronounced between subregions situated in different lobes, with different white matter tracts connecting each subregion^22,23^. On the other hand, an increasing literature has emphasized the presence of large-scale cytoarchitectural gradients across the cortex, suggesting a microstructural differentiation between sensory and transmodal regions as well as long distance similarities in microarchitectural profiles^24,25^. Such large-scale cytoarchitectural gradients can also underlie organization within a subregion such as the mesiotemporal lobe and insula^26,27^. Thereby, fine-grained intraregional differentiation is another important contributor to heterogeneity in the DMN. Fine-grained patterns of differentiation need not be gradients, however. Primate tract-tracing and precision functional imaging studies have revealed interdigitation of connectivity within regions of the DMN, such as the prefrontal cortex and the inferior parietal lobe^28,29^. Thus, while laminar connectivity across the cortex follows consistent rules^30^, microstructure and connections can be organized locally in a range of patterns from relatively smooth gradients to checkered interdigitation. Recent innovations in whole-brain human histology and quantitative in vivo magnetic resonance imaging (MRI) at high fields have made it possible to determine how these various findings manifest in the DMN, enabling the derivation of an anatomically grounded blueprint of its organization.

The microarchitectural make-up of the DMN ultimately influences how it processes information because microarchitecture influences both the intrinsic computation within a region and its connectivity to other regions—the two sides of functional specialization. For instance, the degree of laminar differentiation, which varies in a graded manner across the cortex^24^, reflects different specializations of the underlying cortical microcircuits, ranging from externally focused sensory areas through unimodal and heteromodal cortex to amodal agranular areas^31,32^. Patterns of projections also vary systematically along this gradient^30,33^, forming a hierarchical architecture of cortico–cortical tracts spanning from primary sensory areas to the prefrontal cortex and mesiotemporal lobe^34–36^. Whether a hierarchy constrains connectivity within association cortex (such as the DMN) has been questioned^1,37,38^. Instead, the DMN may comprise densely interconnected yet spatially distributed circuits, operating in parallel to the canonical sensory hierarchies^37^. Distinguishing between hierarchical and nonhierarchical schemas relies upon characterizing how signal flows with respect to the underlying microarchitecture. To this end, state-of-the-art connectivity mapping approaches that emphasize directed signal flow, including recently introduced measures of navigation efficiency (*E*_nav_) of structural connections^39^ and regression dynamic causal modeling (DCM) (rDCM) of functional signals^40,41^, can help adjudicate between different theoretical perspectives. In combination with data-driven microarchitectural mapping, these approaches can elucidate how cortical anatomy constrains the communication of the DMN, shedding light on the perhaps unique organizational principles of human association cortex.

Here, we capitalize on a combination of postmortem histology and multimodal in vivo neuroimaging to map DMN microarchitecture and examine how that microarchitecture contributes to its structural and functional embedding in the brain. In particular, we leverage (1) an established atlas of cytoarchitectural taxonomy (cortical types)^24,42^, (2) whole-brain three-dimensional (3D) histology for fine-grained cytoarchitectonic mapping^43,44^ and (3) multimodal in vivo neuroimaging for approximations of structural wiring and functional flow. Finally, (4) using high-field 7-T MRI, we demonstrate how the discovered relationships between microarchitecture, connectivity and function of the DMN exist within an individual brain.

## Results

### Cytoarchitectural heterogeneity

**Fig. 1:**
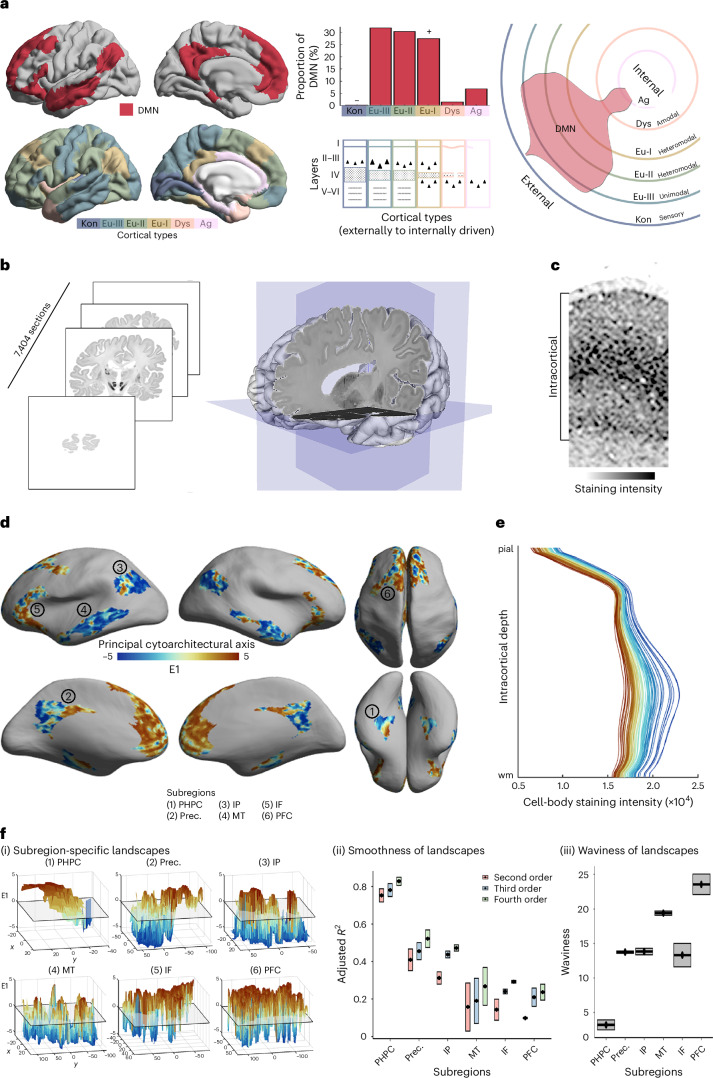
Cytoarchitectural heterogeneity of the DMN. **a**, Distribution of cortical types within the DMN. Upper left, the most common atlas of the DMN^1^ (used in primary analyses) is shown on the cortical surface. Lower left, cytoarchitectonic atlas of cortical types^24,42^. Upper middle, histogram depicting frequency of cortical types within the DMN. The plus sign indicates significant over-representation and the minus sign, under-representation, relative to whole-cortex proportions. Lower middle, schematic highlighting prominent features that vary across cortical types, including the location/size of largest pyramidal neurons (triangles), thickness of layer IV, existence of sublayers in V–VI (gray dashed lines), regularity of layer I/II boundary (straightness of line). Kon, koniocortical; Eul, eulaminate; Dys, dysgranular; Ag, agranular. Right, circular plot representing the spread of the DMN from externally to internally driven cortical types. The percentage of each type within the DMN is depicted by the amount of the respective line (not the area in between lines) covered by the red shaded violin. Similar schematics may be found in extant literature^16,32,89^. **b**, Three-dimensional reconstructed sliced and stained human brain. Coronal slices of cell-body-stained sections (20-μm thick, *n* = 7,404) were reconstructed into a 3D human brain model, BigBrain^43^. **c**, Example cortical patch shows depth-wise variations in cell-body-staining in BigBrain. **d**, Cytoarchitectural differentiation within the DMN. Principal eigenvector (E1) projected onto the inflated BigBrain surface shows the patterns of cytoarchitectural differentiation within the DMN. PHPC, parahippocampus; Prec., precuneus; IP, inferior parietal; MT, middle temporal; IF, inferior frontal; PFC, prefrontal cortex (superior frontal and anterior cingulate cortex). **e**, Cytoarchitectural profiles. Line plots represent cell-body-staining intensity by intracortical depth (from pial to white matter (wm) boundary) at different points along E1. Cortical points with lower E1 (blue) have peaked cellular density in mid–deep cortical layers, indicative of pronounced laminar differentiation, whereas cortical points with higher E1 (red) have more consistent cellular density across cortical layers, illustrating lower laminar differentiation. **f**, Cytoarchitectural landscapes of subregions. (i) Topography of E1 in each subregion shown as 3D surface plots, with E1 as the *z* axis. The *x* and *y* axes are defined by Isomax flattening of each subregion. (ii) Proportion of variance in E1 explained by spatial axes (*x*,*y*) for each subregion and for models of increasing complexity (second- to fourth-order polynomial regression). (iii) Waviness of E1 in each subregion. Upper and lower bounds of each box represent the adjusted *R*^2^ for each hemisphere (*n* = 2), and the center point is the adjusted *R*^2^ averaged across hemispheres.

The DMN is generally agreed to encompass subsections of (1) the parahippocampal cortex, (2) precuneus and posterior cingulate cortex, (3) a caudal region of the inferior parietal lobule, (4) the middle temporal cortex, (5) the inferior fronto-lateral cortex, and (6) a region of the prefrontal cortex, covering primarily the superior frontal gyrus and anterior cingulate, as well as a small part of the middle frontal gyrus^2,3^. Throughout our primary analyses, we used the most common atlas of the default mode network^1^ (Fig. 1a) and identified six spatially contiguous subregions within each hemisphere that correspond to the abovementioned regions (see Supplementary Table 1 for Von Economo areas and Schaefer parcels encompassed by each subregion). In supplementary analyses, we show the replicability of key findings with alternative delineations of the DMN, based on deactivations during externally oriented tasks^15^, independent component analysis of task-based functional MRI (fMRI)^45^, and individualized Bayesian modeling of functional communities^46^.

**Extended Data Fig. 1:**
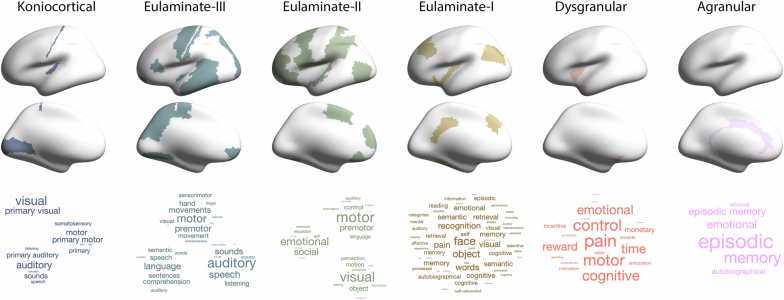
Meta-analytic functional decoding of the cortical type atlas. Meta-analytic functional decoding of the cortical type atlas supports the association, described in literature reviews^43^, between the gradient of cortical types and a shift in function from primary sensory to unimodal to heteromodal to memory-related processes. Using meta-analytic maps of thousands of functional MRI^140^, we extracted terms that were consistently associated with increased activity within the specific cortical type (threshold z-statistic>2). The size of each word reflects the relative strength of its association with the cortical type. Only psychological constructs were retained in the term lists (thus excluding anatomical terms, for example “V1”, and experiment-related terms, for example “healthy controls”). Decoding was performed within spatially contiguous subregions for Kon, Eu-III and Eu-II, because no terms exceeded the threshold when the subregions were combined, due to the distinctive unimodal functions of each subregion.

The most noticeable difference in cytoarchitecture across cortical regions is the degree of laminar differentiation, that is, the distinguishability and thickness of layers. Degree of laminar differentiation is highest in primary sensory areas and decreases along the cortical mantle in a graded manner, reaching a low in agranular cortex, which neighbors hippocampal and piriform allocortex. This gradient of laminar differentiation is synopsized by six cortical types, defined originally by Von Economo^24,42^ (Fig. 1a). Patterns of projections also vary systematically along this gradient^30,33^, forming a hierarchical architecture spanning primary sensory areas to the prefrontal cortex and hippocampus^34–36^. The cortical types (synonymous with levels of sensory hierarchies) are hypothesized to reflect different specializations of the underlying cortical microcircuits, ranging from externally focused sensory areas through unimodal and heteromodal cortex to agranular, paralimbic areas^31,32^. This hypothesized relationship, based primarily on neurophysiological evidence in nonhuman primates and lesion studies in humans^47^, is supported here by meta-analytical decoding of the cortical types, using activation maps from thousands of fMRI studies (Extended Data Fig. 1).

**Extended Data Fig. 2:**
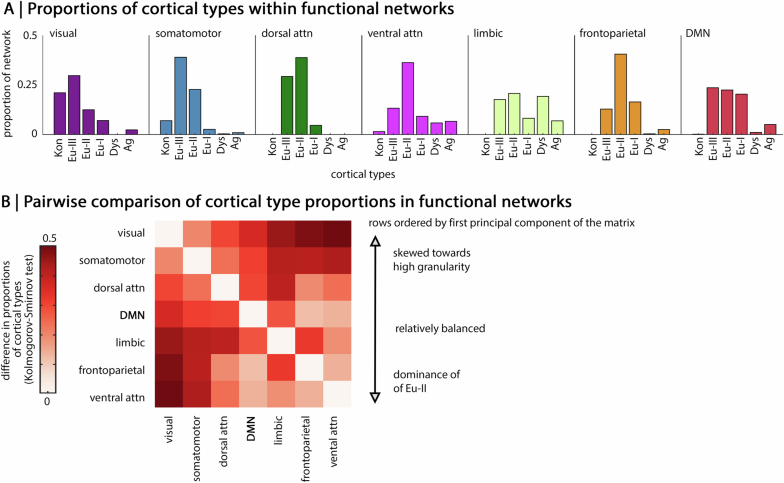
Cortical types and functional networks. **A)** Bar charts illustrate the proportion of cortical types within each functional network **(**for further details, see Supplementary Table 2. **B)** Matrix illustrating the outcome of pair-wise Kolmogorov-Smirnov tests, whereby darker colours reflect greater difference in the cortical type make-up of the functional networks. Rows and columns of the matrix are ordered according to the first principal component, thereby showing that the DMN occupies a middle ground between the functional networks skewed towards high granularity and the functional networks dominated by eulaminate-II.

**Extended Data Fig. 3:**
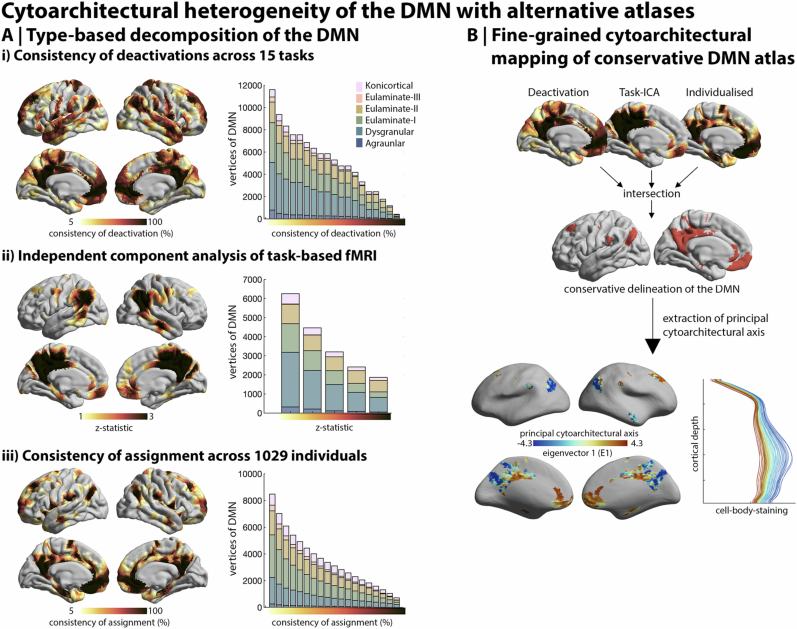
Cytoarchitectural heterogeneity in the DMN replicated with alternative atlases. **A)** The diverse cytoarchitectural composition of the DMN was also evident using alternative atlas definitions. Stacked boxplots illustrate the number of vertices assigned to each cortical type within the atlas with increasingly conservative thresholds for inclusion in the DMN represented along the x-axis. **i)** DMN based on consistency of deactivation during perceptually-driven tasks. Vertex-wise change in the BOLD response were calculated across 787 subjects in Human Connectome Project during fifteen perceptually-driven tasks. Surface projections show the consistency of deactivations (z ≤ -5) across the tasks^20^. **ii)** Association (z-statistic) of each vertex to the DMN derived from an independent component analysis of 7,342 task contrasts^59^. **iii)** Probability of the DMN at each vertex, calculated across 1029 individual-specific functional network delineations^60^. Proportion of the DMN assigned to each cortical type, where the DMN is defined variably based on different consistency thresholds. **B)** Using an intersection of the three approaches in part A, we created a highly conservative delineation of the DMN. Specifically, vertices were included in the conservative atlas if (i) deactivations were observed in more than a quarter of perceptually-driven tasks, (ii) contribution to the task-ICA exceeded a z-statistic of 1 and (iii) assignment to the DMN was observed in more than a quarter of individuals. Subsequently, we replicated the procedure in the primary analysis to extract the principal cytoarchitectural axis. Notably, similar patterns of cytoarchitectural differentiation are evident in this conservative delineation of the DMN. The conservative cytoarchitectural axis also captures a variation from peaked to flat profiles.

Based on overlap of the DMN atlas with a cytoarchitectonic atlas of cortical types^24,42^, we found that the DMN contains five of six cortical types (Fig. 1a). This make-up was distinctive relative to other functional networks (Extended Data Table 1, all Kolgomorov–Smirnoff tests >0.11; *P* < 0.001). Indeed, pairwise comparisons showed that all networks exhibited a unique composition of cortical types (Extended Data Fig. 2). Notably, of all functional networks, the DMN contains the most balanced representation of the three eulaminate types commonly associated with processing of sensory information and its progressive integration (eulaminate-I, -II and -III). In addition, the DMN contains dysgranular and agranular cortex that are often linked to internally generated processes such as memory and affect^32^ (Extended Data Fig. 1). These cortical types are not represented equally within the DMN, however (*χ*^2^ = 1,497; *P* < 0.001). Approximately 90% of the DMN is eulaminate, which is even higher than the cortex-wide rate of 84% (Extended Data Table 1). To evaluate whether this type of cortex is over-represented in the DMN, we compared the proportion of cortical types within the DMN and within 10,000 rotated versions of the DMN. The rotated versions are generated by randomly spinning the functional network atlas on a spherical representation of the cortex, providing a null distribution of outcome statistics that account for the network’s size and distribution. In doing so, we found that the DMN over-represents eulaminate-I (18% increase; *P*_spin_ = 0.006), classically known as heteromodal cortex, which is hypothesized to process information from several sensory domains^32^ (Extended Data Fig. 1). This distinctive composition of cortical types was evident regardless of slight alterations to the DMN atlas (Extended Data Fig. 3). The broad range of cortical types in the DMN, combined with the over-representation of eulaminate-I, is consistent with a role of this network in integration of information from several systems, including those linked to sensory and memory processes.

**Extended Data Fig. 4:**
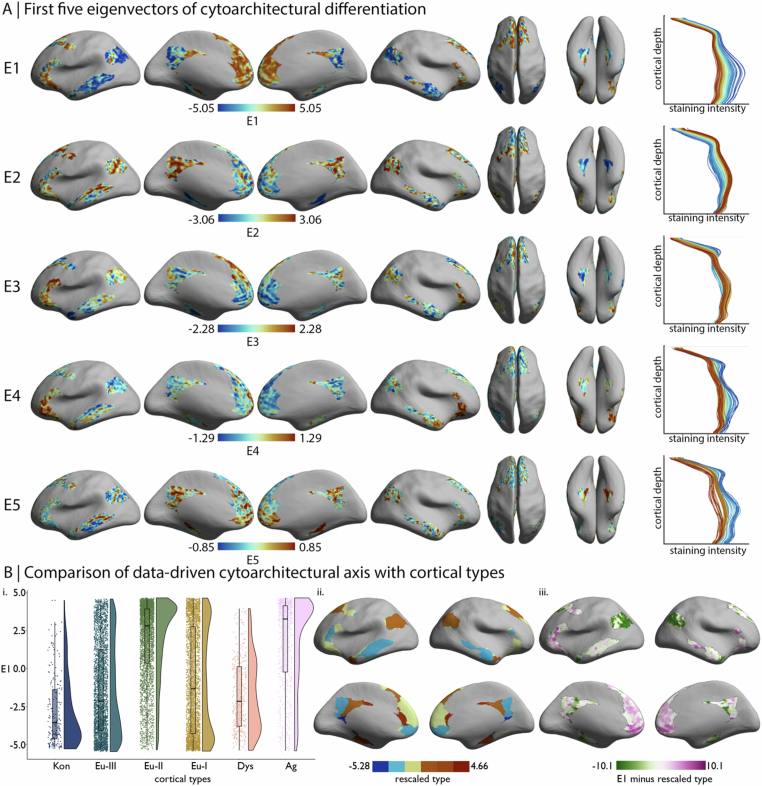
Lower-order eigenvectors and comparing E1 to cortical types. **A)** First five eigenvectors projected on the inflated BigBrain surface. For line plots on the right, staining intensity profiles were averaged within 100 bins of the respective eigenvector and coloured by eigenvector position. **B) i**. Raincloud- and box-plots show the distribution of E1 across cortical types (n = 109/3785/3982/2913/282/669 for Kon/Eu-III/Eu-II/Eu-I/Dys/Ag). Box plots represent minimum, quartile 1, median, quartile 3 and maximum. **ii**. Cortical type assignment (1:6) was rescaled to the range of E1 then subtracted from E1, producing a deviation map that highlights where the type-based and data-driven depictions of DMN cytoarchitecture differ. Negative values indicate lower E1 than expected by a linear relationship with cortical type, whereas positive values indicate higher than predicted E1. Thus, the E1 pattern is distinct to the gradient of laminar elaboration that is captured by the cortical types. Both are anchored by koniocortex on one side and agranular cortex on the other, but they differ in the ordering of Eu-and dysgranular areas.

Having established that the DMN contains a broad array of cortical types, we next adopted a data-driven approach to characterize fine-grained spatial patterns of cytoarchitectural variation. We transformed the functional network atlas^1^ to a 3D cell-body-stained postmortem human brain^43^ using specially tailored cortical registration procedures^44,48^. Using intracortical profiles of cell-body-staining intensity (Fig. 1c,e), we assessed cytoarchitectural variability within the DMN, mapping cytoarchitectural variation by nonlinear manifold learning^49^ (Fig. 1d and Extended Data Fig. 4). The first eigenvector (E1) of this manifold, hereafter referred to as the cytoarchitectural axis, described a shift in the shape of the underlying cytoarchitectural profiles from peaked to flat (Fig. 1e) and reflects variations in cellular density (Fig. 1c). The cytoarchitectural axis is anchored on one end by unimodal eulaminate-III cortex (for example, retrosplenial and posterior middle temporal) and on the other by agranular cortex (for example, medial parahippocampus and anterior cingulate). Thus, the endpoints of the cytoarchitectural axis are the most extreme cortical types found within the DMN (Extended Data Fig. 4). Beyond the endpoints, however, the cytoarchitectural axis deviates from the gradient described by cortical types^24,25,32^ (Extended Data Fig. 4). This pattern does not discriminate subregions of the DMN or follow an anterior–posterior gradient as seen in neuronal density^50^. Instead, we observed a mosaic of different spatial topographies across DMN subregions, where neighboring points are sometimes distinct and distant points are sometimes similar. Our data-driven approach thus indicates that organization within the DMN is unlike those observed across sensory hierarchies and is relatively unconstrained by large-scale spatial gradients^37,38^.

**Extended Data Fig. 5:**
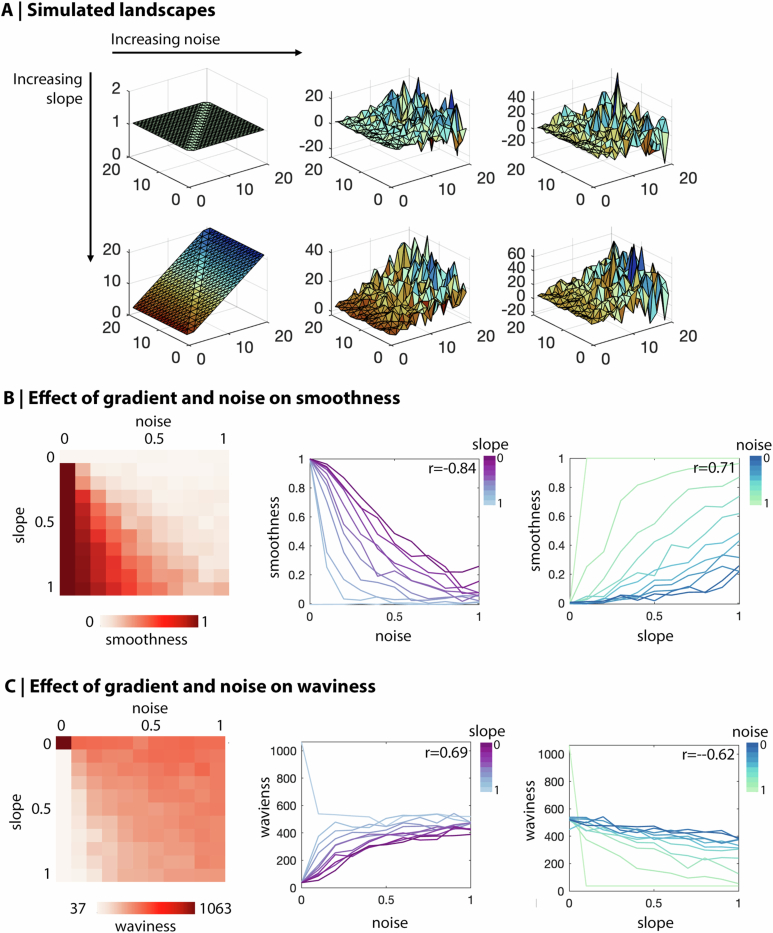
Landscape simulations of smoothness and waviness. As expected, smoothness decreases with noise and increases with slope, whereas waviness increases with noise and decreases with slope. **A)** We simulated 121 landscapes with varied slopes and bumpiness (noise). x and y values were identical in all landscapes, while the z-axis – reflecting E1 topography in the main study – was modulated in each simulation. The z-axis value was calculated as “(x * *slope*) + (*rand* * *sigma*)”, where *slope* is a value within [0:0.1:1], *rand* is a vector of normally distributed pseudorandom numbers the length of x and *sigma* is the product of x and a value within [0:0.1:1]. **B-C)** ***Left****.* Each square of the matrix represents a simulated landscape, with rows reflecting increasing slope and columns reflecting increasing noise. ***Centre-Right****.* Line plots show the outcome metrics of simulations per row and column, respectively. r-values represent the outcome of partial product-moment correlations (for example correlation of smoothness with noise, controlling for slope).

A closer look at the topography of cytoarchitecture highlights the (dis)similarity of neighboring areas within the DMN. Given the ubiquity of local connectivity in the cortex, topography provides important information on the form of communication within spatially contiguous subregions. Subregions of the DMN evidently vary in terms of their cytoarchitectural topography (Fig. 1f), and we quantified these differences using two complementary measures: smoothness and waviness. The smoothness of the microarchitectural landscape was calculated by evaluating the proportion of variance in the cytoarchitectural axis that could be accounted for by spatial axes. Waviness was indexed by deviations from the mean—a common technique in mechanical engineering^51^ (see Extended Data Fig. 5 for simulation-based validation of these metrics). We found that subregions differ significantly in terms of both smoothness and waviness (smoothness (second-/third-/fourth-order, *F* = 14.5/14.9/20.1; *P* < 0.004; waviness, *F* = 48.3; *P* = 0.001). Smoothness is particularly high in the parahippocampus, showing that its cytoarchitectural axis follows a relatively smooth gradient, as shown previously^27,52^. Conversely, the prefrontal cortex exhibits especially high waviness. This pattern of frequent changes across the cortex, back-and-forth between two contrasting properties, is reminiscent of the interdigitated connectivity patterns that are known to exist within the prefrontal cortex^28,29,53^. This analysis establishes that the DMN contains distinct cytoarchitectural patterns representative of two different ways that neural signals are hypothesized to be integrated in the cortex: a mesiotemporal gradient associated with progressive convergence of information^54,55^, and prefrontal interdigitation that enables information from disparate sources to be linked^28^. Together, these metrics, further described and validated in Extended Data Fig. 5, quantify how cytoarchitectural landscapes vary between subregions, from a relatively simple gradient in the parahippocampus, well-explained by the spatial regression model and with low waviness, to marked fluctuations in the dorsal prefrontal cortex, characterized by high waviness and poor regression model performance.

### Receivers on the periphery and an insulated core

Next, using multimodal MRI, we explored how the variable cytoarchitecture of the DMN relates to its connectivity. We hypothesized that connectivity would covary with the cytoarchitectural axis (E1, Fig. 1d), because propensity for connectivity increases with cytoarchitectural similarity. Although this principle has been observed across association and sensory regions^30,33^, it remains unclear how it applies specifically to the DMN.

**Fig. 2:**
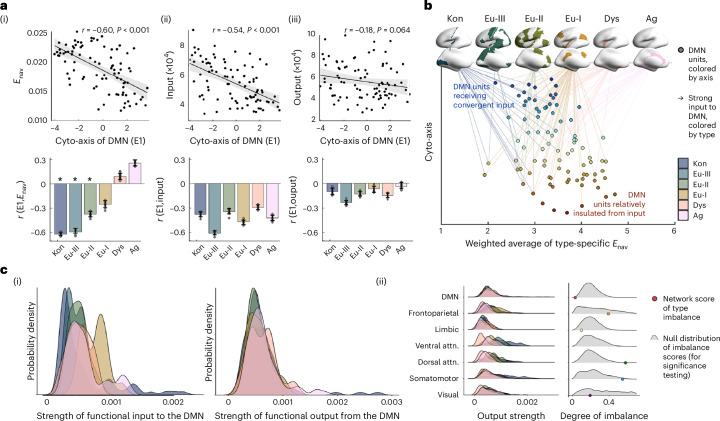
Organization of DMN connectivity. **a**, Top, scatterplots show correlation of cytoarchitectural (Cyto-axis) axis (E1) with average (i) structurally modeled *E*_nav_, (ii) functionally modeled input and (iii) functionally modeled output. Each point represents a node of the DMN; *r* and *P*_uncorrected_ values indicate the statistical outcomes of two-sided product–moment correlation tests (subregion assignment is illustrated in Extended Data Figs. 6a and 7 and Supplementary Table 1). Line plots are presented with 95% confidence interval shading. Bottom, bar plots shows the linear correlation coefficient (*r*) of E1 with average connectivity to each cortical type. The stability of the correlation coefficient was calculated by repeating the procedure ten times, each including 90% of datapoints. Error bars, s.d. of the *r* value across repetitions. Asterisks, significant negative *r* values indicating that DMN nodes with peaked profiles have higher *E*_nav_ with externally driven cortical types and stronger input from most cortical types. **b**, Multimodal model of DMN organization shows the dual character of the DMN, including areas with convergent input and insulated areas. All points in the scatterplot represent units of the DMN, are colored by position along the cytoarchitectural axis (*y* axis) and are organized along the *x* axis based on weighted average of type-specific *E*_nav_. The top 75% of functionally defined inputs are shown. **c**, The DMN is unique amongst functional networks in balancing the strength of output across cortical types. (i) Distributions of strength of input from and output to each type. Colored ridge plots show probability distributions of connectivity between the DMN and each cortical type. For functional output, the DMN exhibits overlapping, normal distributions, whereas for functional input, type-wise differences are evident. (ii) Comparing networks in terms of balance of their output per type. Focusing on functional output, colored ridge plots show distributions for all networks, illustrating more balance between types in the DMN. Right, Imbalance of connectivity to distinct cortical types evaluated as the KL divergence from a null model with equal connectivity to each type. Colored dots show the empirical KL divergence for each network and the gray density plots show the null distribution of KL divergence values based on 10,000 spin permutations. Permutation testing indicated that the DMN is unique among functional networks in balancing output across cortical types (that is, imbalance lower than 95% of permutations). attn., attention network.

**Extended Data Fig. 6:**
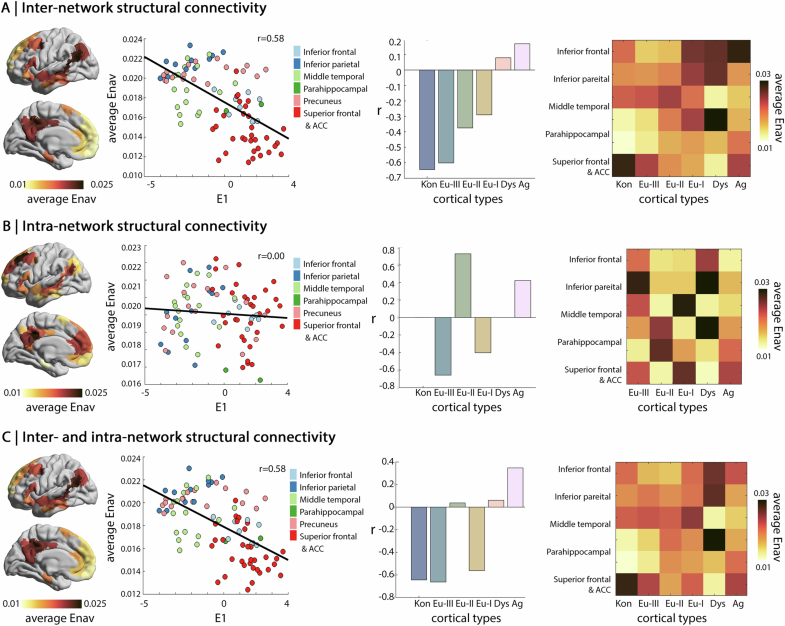
Intra- and inter-structural connectivity of the DMN with respect to the cytoarchitectural axis (E1) and cortical type. Variations in navigation efficiency as a function of the cytoarchitectural axis within the DMN, DMN subregion and cortical type. Panel **A**) involves connections from each node of the DMN with all nodes outside the DMN (as in the primary analysis), Panel **B**) connections from each node of the DMN to all other nodes of the DMN and Panel **C**) connections from each node of the DMN to all other nodes. *Far left*. Cortical maps show average navigation efficiency. *Centre left*. Scatterplots show the correlation of the cytoarchitectural axis (E1) with average navigation efficiency, with points coloured by the seed parcel’s position within the DMN. *Centre right*. Bar plots show the linear correlation coefficient (r) of E1 with average navigation efficiency to each cortical type. *Far right*. Matrix shows the average navigation efficiency between each subregion of the DMN and each cortical type.

First, we measured communication efficiency along white matter tracts^39^ using diffusion MRI tractography^56^. Navigation is a decentralized communication strategy that is particularly suited to spatially embedded networks, which has recently been proposed to study structural connectivity and structure–function relationships in the human brain^56^ (see Methods for further description and motivation). We found that the propensity to communicate with other cortical areas (indexed by average *E*_nav_^39^) varied within the DMN (coefficient of variation = 18%). Areas toward one end of the cytoarchitectural axis of the DMN, specifically those with more peaked cytoarchitectural profiles, such as the anterior cingulate and more anterior aspect of the precuneus, exhibited more efficient communication with the rest of the cortex (*r* = −0.60; *P*_spin_ = 0.001; Fig. 2a(i)). This effect was particularly pronounced for communication with perceptually coupled cortical types (koniocortical/eulaminate-III/eulaminate-II; *r* = −0.63/−0.60/−0.38, *P*_spin_ < 0.025; Fig. 2a(i)). Thus, the cytoarchitectural organization of the DMN also correlates with spatial patterns of tract-based communication, especially between the DMN and cortical areas engaged in sensory processing. This pattern of covariation was specific to connectivity between the DMN and non-DMN areas, and did not apply to connectivity within the DMN (Extended Data Fig. 6), suggesting that inter- and intranetwork connectivity may involve distinct rules of organization that are embedded within in more general, cortex-wide principles, such as the structural model^30^.

**Extended Data Fig. 7:**
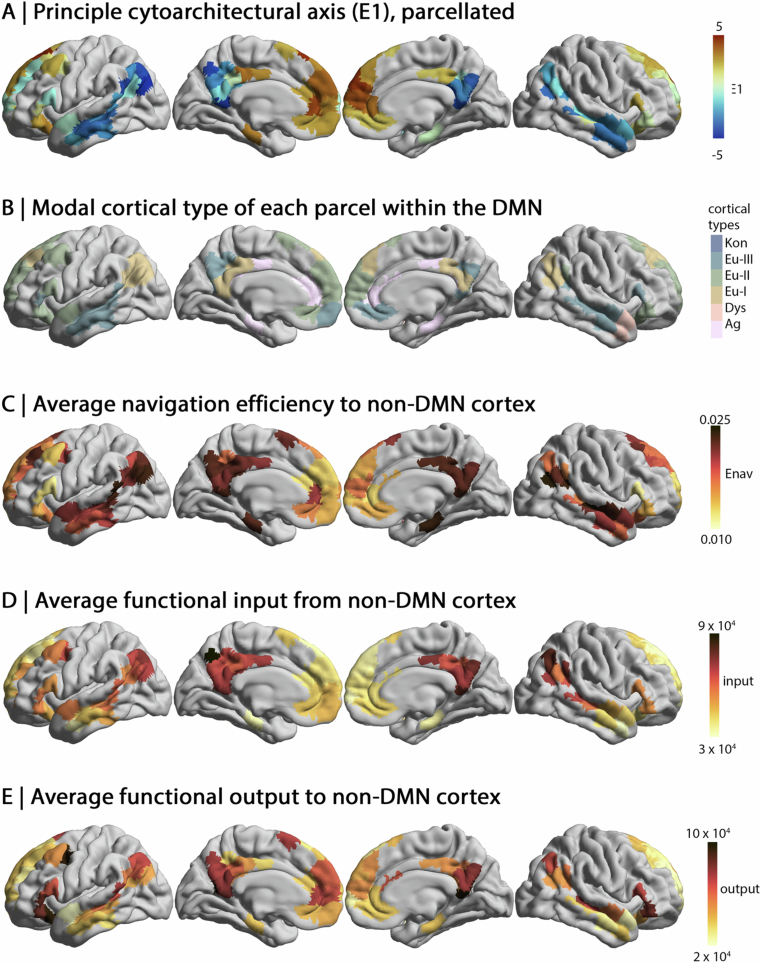
Overview of key cortical maps. Cortical maps illustrate the key axes of variation in **A**-**B**) cytoarchitecture, **C**) structural connectivity and **D**-**E**) signal flow. Exact values for each parcel can be found in Supplementary Table 1.

Next, we examined the consequences of this structural organization on the functional flow of information in the cortex. We applied rDCM—a scalable generative model of effective connectivity^40^ to resting-state fMRI timeseries of 400 isocortical parcels, covering the entire isocortex (Methods). In the current work, we conducted a whole-cortex rDCM, then selected DMN parcels as targets for functional input analyses and DMN parcels as seeds for functional output analyses. Functionally estimated input and output varied within the DMN (coefficient of variation = 24% and 29%, respectively). Average strength of input was significantly higher to those areas of the DMN with more peaked cytoarchitectural profiles (*r* = −0.54; *P*_spin_ < 0.001), that is, those regions that were also highlighted as having more efficient communication with the rest of the cortex in the above structural connectivity analysis (see Extended Data Fig. 7 for a comparison of cortical maps). Examination of type-specific connectivity showed limited discrimination between cortical types, whereby inputs from externally and internally focused cortical types were all concentrated on DMN areas with peaked cytoarchitectural profiles (Fig. 2a(ii),(iii) and Supplementary Table 2). Thus, several inputs converge on a subset of DMN subunits, such as inferior parietal and precuneus areas, whereas a subset of DMN subunits—those with flat cytoarchitectural profiles—remained relatively insulated from cortical input. Output did not covary with the cytoarchitectural axis (*r* = −0.18; *P*_spin_ = 0.064; Fig. 2a(ii),(iii)). These findings were consistent in a replication dataset and when including subcortical structures and the hippocampus in the model (Supplementary Table 2). Together, these analyses suggest that the DMN comprises two microarchitecturally distinct subsets—one with highly efficient tract-based communication with cortical areas implicated in perception and receiving convergent input from across all levels of sensory hierarchies, and another that exhibits less efficient tract-based communication with the rest of the cortex and is relatively insulated from input signals from sensory systems (Fig. 2b).

### A unique balance of output

Focusing on the anatomy of the DMN revealed its distinctive pattern of cytoarchitectural heterogeneity, which constrains how it communicates with other systems. Now, we turn our attention to how these anatomical properties contribute to the position of the DMN in large-scale cortical organization by understanding how effective functional connectivity of the DMN is distributed across cortical types.

**Extended Data Fig. 8:**
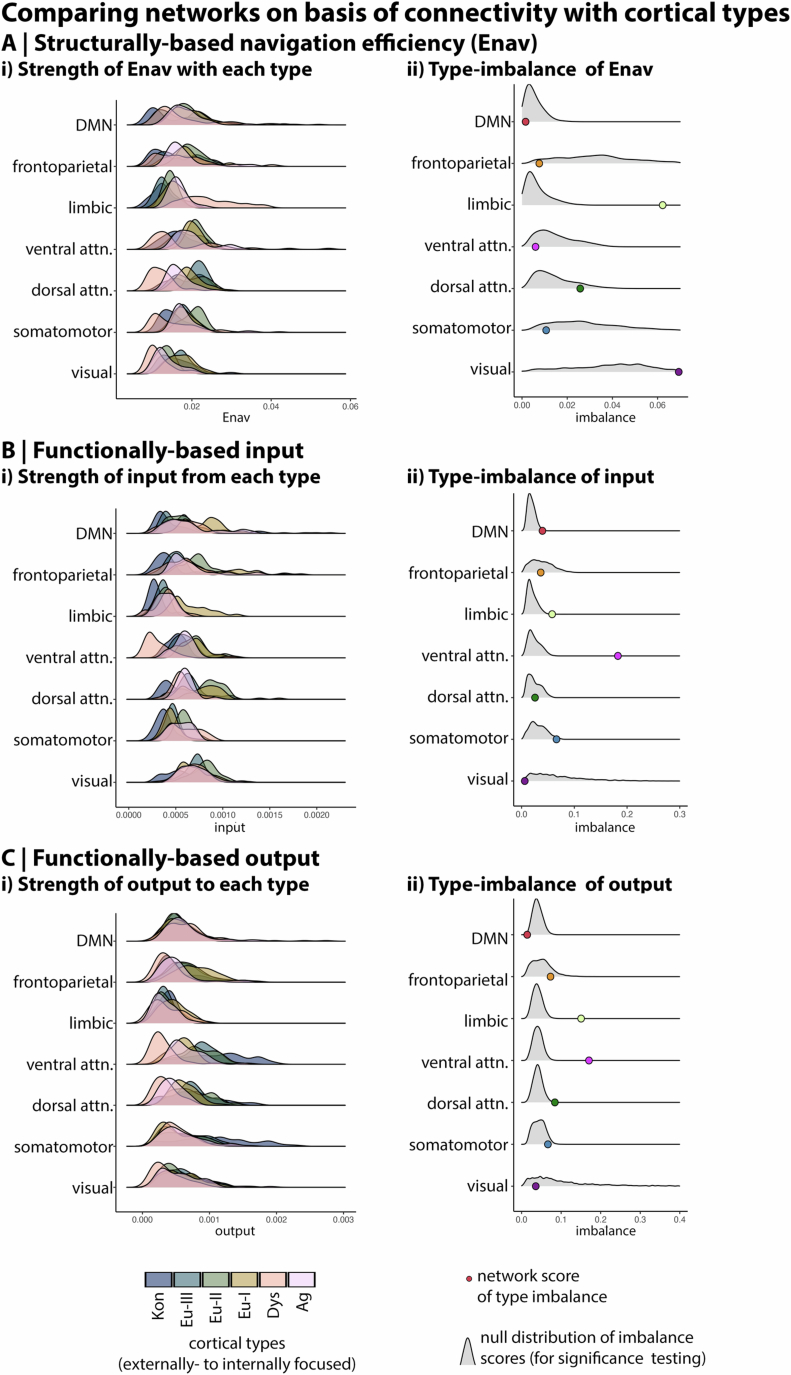
Comparison of functional networks based on inter-network connectivity to different cortical types. Coloured ridge plots on the left of each panel show probability distributions of connectivity between the functional networks and non-DMN cortical types. We evaluated the imbalance of connectivity across cortical types using the Kullback-Leibler (KL) divergence from a null model with equal connectivity to each type. On the right of each panel, coloured dots show the empirical KL divergence for each network and the grey density plots show the null distribution of KL divergence values based on 10,000 spin permutations. **A)** The DMN exhibits the most balanced navigation efficiency across cortical types, compared to other functional networks. The balance of the DMN did not reach a level of significance relative to spin permutations, but spin permutations account for the size and distribution of the network, thus we may infer it is the large size and wide distribution of the network that enable the DMN to strike a balance in communication across cortical types. **B)** Input to the DMN is not balanced with regards to cortical types. Stronger input comes from heteromodal, Eu-I cortex, which aligns with the over-representation of this cortical type within the DMN. **C)** The DMN is unique amongst functional networks in exhibiting balanced output to all cortical types, which is further supported by the balance of the DMN reaching significance in spin permutation testing.

First, we discovered that the DMN communicates in a balanced manner with all cortical types. Compared with other functional networks, the DMN exhibits the most balanced efficiency of communication across cortical types (that is, lowest KL divergence from null model (Extended Data Fig. 8 and see Supplementary Table 3 for statistics)). We could further specify that output of the DMN is balanced across the cortical types, but input is not (Fig. 2c(i) and see Supplementary Table 3 for statistics and replication). In other words, the DMN outputs signals in approximately equal strength to all cortical types (that is, all levels of sensory hierarchies). Of all the functional systems in the human cortex, only the DMN exhibited this balance in output across cortical types (Fig. 2c(ii)). The spatial distribution, internal heterogeneity and connectivity of the DMN thus engender a unique ability to receive temporally distinct signals and then send neural signals that influence all levels of the sensory hierarchies in a similar manner.

### Correspondence of microarchitecture and connectivity within an individual brain

**Fig. 3:**
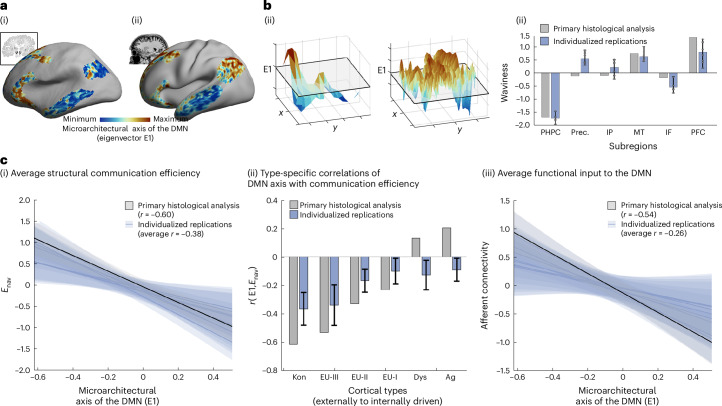
Replication of crossmodal analyses within individual brains using 7-T MRI. **a**, Comparison of microstructural axes. The principal eigenvector of microstructural variation in the DMN (E1) was extracted from myelin-sensitive qT1 MRI^57^, in line with the procedure employed on the histological dataset (BigBrain), revealing similar patterns. (i) Primary histological axis, (ii) MRI-derived axis. **b**, Subregions differ in MRI-derived microstructural axis topography. The roughness of MRI-derived microstructural differentiation varied between subregions in line with histological evidence. (i) Parahippocampal (left) and prefrontal (right) landscapes. (ii) Landscape waviness. The parahippocampus exhibited a graded transition from high-to-low E1, reflected by high smoothness and low waviness, whereas the prefrontal cortex exhibited an undulating landscape with high waviness. For individual replications (*n* = 8), bars show the median across subjects and error bars depict the maximum and minimum. **c**, Communication efficiency and functional input decrease along the microstructural axis of the DMN. Using individual-specific measures, we consistently found that cortical points with higher E1 were associated with (i) lower average *E*_nav_, (ii) especially lower *E*_nav_ with perceptually coupled cortical types and (iii) lower functional input. Line plots are presented with 95% confidence interval shading. Column plots with error bars, for individual replications, show mean ± s.d. across subjects (*n* = 8).

To demonstrate that our findings generalize to individual brains, we acquired high-resolution quantitative T1 (qT1) relaxometry MRI, alongside diffusion-weighted and functional MRI in eight healthy people using a 7-T MRI. Methods were identical to those described above, except that histology was replaced by qT1. We hypothesized that qT1, sensitive to cortical myelin^57,58^, could recapitulate regional differences in cytoarchitecture, because cortical areas and intracortical layers defined on cyto- or myeloarchitecture align^59^, and our previous work has shown strong correspondence of principal axes of microstructural differentiation derived from histology and qT1 MRI^25^. While the qT1 and histological datasets differ in terms of biological sensitivity (myelin versus cell bodies) and resolution (500 μm versus 100 μm), the patterns of microarchitectural differentiation in the DMN had moderate similarity between the modalities (*r*_avg_ = 0.34; *P*_avg_ < 0.001), for example, highlighting microstructural differences of the prefrontal cortex from the lateral temporal region (Fig. 3a). We also repeated the analysis using individual-specific DMNs (Methods) and found highly similar axes (Supplementary Fig. 1). Thereby, microstructural variation within the DMN is not due to idiosyncratic positioning of the DMN, relative to the group-average atlas.

Although idiosyncrasies and crossmodal differences were evident, especially in the lateral parietal and anterior cingulate regions (Supplementary Fig. 1), the topography of microarchitectural differentiation was similar in both qT1 and histological datasets, varying from a smooth gradient in the mesiotemporal lobe to higher waviness in the prefrontal cortex (Fig. 3b). Indeed, subregion smoothness (*r*_avg_ = 0.51; *P*_avg_ = 0.09) and waviness (*r*_avg_=0.74; *P*_avg_ = 0.011) were correlated between the datasets. Furthermore, in line with our primary analyses, communication efficiency between DMN subregions and the rest of the cortex was higher toward one end of the microstructural axis (*r*_avg_ = −0.38; *P*_avg-spin_ = 0.015). This effect was especially pronounced with regards to communication to perceptually coupled cortical types (koniocortical/eulaminate-III: *r*_avg_ = −0.40/0.37; *P*_avg-spin_ = 0.044/0.089). Finally, functional input also tended to decrease along the microstructural axes (*r*_avg_ = −0.26; *P*_avg-spin_ = 0.101). Together, these individual-level analyses indicate that the microarchitectural axis of the DMN discriminates a zone of multimodal convergence from a core that is relatively insulated from external input (Fig. 3c). Thus, in line with histological evidence, the MRI-based approach highlights that a subsection of the DMN is relatively insulated from external input.

## Discussion

Historically, anatomical details of brain systems have helped constrain accounts of their function^36,60^. Our study extended this perspective to the DMN—one of the most extensively studied yet least well understood systems in the human brain. Leveraging postmortem histology and in vivo MRI, we observed pronounced cytoarchitectural heterogeneity within the DMN, showing that the network encompasses types of microarchitecture variably specialized for modality-specific, heteromodal and self-generated processing^24,32^. By combining cytoarchitectural information with structural and functional connectivity, we found that the DMN contains convergence zones that receive input from other cortical regions, as well as a relatively insulated core. Moreover, unlike other functional networks, outgoing signals of the DMN are of similar strength to different cortical types, meaning the network may be uniquely capable of influencing function across all levels of sensory hierarchies in a relatively coherent manner.

### The DMN harbors a complex landscape of cytoarchitecture and connectivity

Complementary theory- and data-driven analyses revealed the heterogeneous cytoarchitecture of the DMN. First, comparison of functional and cytoarchitectural atlases showed that the DMN contains a wide range of cortical types, from eulaminate-III to agranular. This type-based analysis demonstrates the extent of cytoarchitectural variation within the DMN and that it spans several steps of laminar elaboration^24,42^. Second, applying nonlinear dimensionality reduction techniques to a 3D histological reconstruction of a human brain highlighted an axis of cytoarchitectural differentiation, E1, within the DMN that is distinct from the gradient of laminar elaboration. Both the type-based and data-driven axes stretch between the primary sensory areas and the allocortex, but they capture different aspects of cytoarchitectural similarity in eulaminate-II, -I and dysgranular cortex. For instance, while cortical types are related to the combination of qualitative and quantitative measures across cortical layers, the most prominent differences pertain to neuronal density in layers II/III (ref. ^61^). In contrast, the first data-driven axis is related primarily to cytoarchitectural markers in the mid-to-deep cortical layers. Higher-order components, such as E4 and E5, may better reflect the cytoarchitectural features captured by cortical types, and further research is necessary to address the sensitivity of our automated profiling approach to superficial versus deeper layers (for example, the influence of cortical curvature, resolution and interpolation techniques), especially given the singular nature of the BigBrain dataset. In addition, cortical types are defined by topology, that is, their spatial relations, whereas the data-driven axis is derived in a manner that is agnostic to spatial constraints. The latter approach revealed pronounced cytoarchitectural variation within the DMN that is not as constrained by cortex-wide gradients, but rather involves a complex pattern of subregion-specific cytoarchitectural topographies, including both local gradients and interdigitation.

A core principle of neuroanatomy holds that topographies of cortical microstructure, connectivity and function are intrinsically related^47,62–64^. We found a clear example of this relationship in the DMN, whereby the principal cytoarchitectural axis captures differences in structural and functional connectivity to other cortical territories. By combining diffusion-based tractography with physical distance measurements into a model of *E*_nav_^39,56^, we found that the strength of communication between the DMN and other cortical areas was related to the cytoarchitecture of each endpoint. Specifically, regions of the DMN low on E1 exhibited preferentially higher *E*_nav_ to granular cortical types. Tract-tracing studies in macaques focusing on circumscribed regions of the DMN, such as the precuneus/posterior cingulate, have shown similar patterns of differential connectivity to primary sensory areas^65,66^. The influence of E1, rather than cortical types, in our analyses, suggests that unique principles of cortical organization may apply specifically to internetwork connectivity of the DMN.

Repeating the analysis with whole-brain effective connectivity^40^, we observed decreasing afferent connectivity along the principal cytoarchitectural axis E1. Areas of the DMN with high afferent connectivity, such as the precuneus and inferior parietal lobe, probably have more supragranular neurons than areas with low afferent connectivity, such as the anterior cingulate and superior frontal gyrus^67,68^. It is possible, therefore, that regions that act as receivers within the DMN may be especially important in feedforward processing^69,70^. This pattern suggests that preferential *E*_nav_ from certain subunits of the DMN to more granular types may relate to the speed or directedness of communication, especially given that more granular areas exhibit faster intrinsic timescales^71–73^ and sensory areas require high-fidelity information^32^. In contrast, parcels of the DMN with flatter profiles (that is, higher E1) are more insulated from primary sensory areas (also evident in ref. ^19^) and receive less input from non-DMN cortex. This suggests that the characterization of the DMN as distant from input^11^ is especially true for those insulated subsections of the DMN (for example, the anterior cingulate). The degree of insulation may be concordant with suppression during externally oriented tasks, which is also regionally variable within the DMN^74^. In line with our results, subunits of the DMN high on E1, such as the medial prefrontal cortex, are suppressed for longer than those lower on E1, such as the temporoparietal junction. Taken together, the connectivity analyses, therefore, illustrate the complementary functional roles of cytoarchitecturally distinct subunits of the DMN, from receivers on one side of the cytoarchitectural axis to insulated subunits on the other side.

### Translation from postmortem to in vivo research

Our main analyses combined postmortem histology from one person with in vivo imaging in different populations of healthy people. As such, structure–function relationships may be influenced by crossmodal registration as well as interindividual differences. In this regard, our replication analysis using 7-T MRI shows that fine-grained insights into microarchitecture, connectivity and function persist at an individual level and are observable in vivo. Nevertheless, some observed differences between the histological and MRI axes may be related to several factors including modality (cyto- versus myeloarchitecture), tissue type (postmortem versus in vivo) or interindividual variation. Further work with several modalities acquired in a single brain (for example, MRI and histology or cyto- and myelostaining) is necessary to determine the source of these differences. Extending these methods to in vivo imaging opens unprecedented possibilities to formally test anatomically grounded hypotheses of the role of the DMN in cognition and behavior. For example, the present multimodal model of the DMN could be combined with psychometric data and experience sampling to test how changes in the DMN impact cognitive performance, thought processes and action. Such modeling is a critical next step in evaluating the causal role of the DMN in the brain, as well as the source of its cofluctuations (for example, by studying the role of neuromodulatory systems).

### The DMN and cortical hierarchies

Our investigation of DMN microarchitecture can also help discern the network’s relationship to cortical hierarchies. Established by foundational research in nonhuman animals and increasingly confirmed in the human brain, hierarchies are a recurring motif in cortical organization^34,75^. In general, hierarchical architectures are related to inter-regional variations in temporal dynamics^71,73^ and neural representations. Hierarchies in sensory cortex are well documented^36^, in part because their properties can be confirmed directly through the stimulation of sensory systems. Hierarchies in association networks, on the other hand, are more challenging to determine^37^, due in part to difficulties in determining a ground truth for their ‘bottom’ and ‘top’. In lieu of such functional evidence, our microarchitectural findings are important because they show the DMN entails two properties of hierarchies: (1) connectivity organizable by distinct levels and (2) the existence of an apex that is relatively insulated from external input. Unlike sensory hierarchies, however, which increasingly intersect at upper levels, the internal organization of the DMN is less constrained by spatial gradients and exhibits more balanced interfacing with several levels of sensory systems as well as the limbic system. By expanding the conceptualization of hierarchies beyond sensory systems, our study helps illuminate the diverse nature of information processing in the brain, which is likely to be important in understanding the mechanisms that underpin the role of the DMN in human cognition and action.

Our conceptualization of the DMN as an association hierarchy expands upon previous ideas, such as the DMN as the apex of Margulies et al.^19^ or as a parallel network to the sensory-fugal hierarchy of Buckner and Krienen^37^. Certain features of these theories are concordant with our results, such as (parts of) the DMN being insulated from input and the distinctiveness of information processing in the DMN. However, our analyses demonstrate that connectivity is organized along the most prominent cytoarchitectural axis of the DMN, which is neither nested within nor parallel to the sensory-fugal hierarchy. Instead, the DMN seems to protrude from the sensory-fugal hierarchy, with strong afferent connectivity on one end and insulation on the other. The areas with convergent afferents, as well as connections within the DMN, may enable the recombination of neural processes that would not be possible within sensory-fugal processing streams^36^. Such topological complexity is thought to be an important trade-off in development and evolution of biological neural networks^76^ and illustrates how the DMN can play a distinctive role in information integration as an association hierarchy.

### Understanding the role of the DMN in cognition and action

We close by speculating on how our analysis can constrain accounts of the contribution that the DMN makes to human cognition and action. Our study suggests several anatomically grounded hypotheses on how the DMN contributes to a broad range of cognitive states. For instance, the topography of cytoarchitecture can shed light on the different forms of information integration, because more than 90% of cortico–cortical connections are between neighboring microcircuits^77^. We observed microarchitectural gradients in the mesiotemporal subregion—a pattern linked previously to sequential transformation of signals from low- to higher-order representations^19,78^ and a gradual shift in functional connectivity from the multiple-demand network to fronto-temporal pole areas^27,79^. In contrast, the interwoven layout of different types of microarchitecture within prefrontal subregions, perhaps related to interdigitation of connections^28,53^, may provide a structural substrate to support domain specialization^29,80^ and cross-domain integration^28^. Understanding the complex cytoarchitectural topography of the prefrontal cortex may also help to understand the region’s functional diversity, which involves both subregional specialization and functions that are ‘greater than the sum of its parts’^81^. The presence of both graded and interdigitated motifs within the DMN suggests that, when these regions function as a collective, they could contribute to brain function in a manner that combines two different types of integration. Furthermore, associations between external and internal modes of cognition and the DMN may be explained by shifting the functional balance from input-oriented to more insulated regions. Such a mechanism would also align with functional imaging studies showing regional differentiation within the DMN for different tasks^82,83^, such as reading versus mind-wandering^84^, which in turn could be linked to how different regions of the DMN participate in or cross-talk with other networks^10,85^. In light of the dynamic reconfiguration of functional networks across cognitive states^86^, it will be important to extend the present analysis approach to study the structural properties of the DMN across several functional contexts. Additionally, the unique balance that the DMN strikes in terms of its functional output across cortical types may help to unify neural activity across brain systems or verify predictions of the world against memory in real time^15,87^.

Taken together, our study offers a set of anatomical hypotheses on how the human brain may enable the formation of abstract representations and uses these to inform cognition across a range of domains. Specifically, the functional multiplicity of the DMN is pillared upon its internal heterogeneity, possession of receivers and more insulated subunits as well as its balanced communication with all levels of sensory hierarchies. This set of unique features outlines an anatomical landscape within the DMN that may explain why the DMN is involved in states that cross traditional psychological categories and that can have opposing features.

Since its conceptualization, the DMN has been marked by controversy. Various approaches produce the DMN, which has led to a certain ontological capaciousness, that is, there is a degree of blurriness about what the DMN is and how to define it^88^. Our study suggests that blurriness of the DMN in both spatial and conceptual terms may be explained by variation in microstructure within subregions and their unique connectivity to other regions of cortex. Specifically, the DMN may take on different forms of cognition by recruiting different parts of each subregion, while the broader system maintains the ability to broadcast coherent signals to the rest of the brain. It is possible that the capacity for a set of distributed functionally diverse brain regions to operate in a coherent manner may be a core feature of how brain function supports the range of different behaviors that we as a species are capable of engaging in.

## Methods

### Histological data

An ultrahigh-resolution 3D reconstruction of a sliced and cell-body-stained postmortem human brain from a 65-year-old man was obtained from the open-access BigBrain repository on 1 September 2020 (https://bigbrain.loris.ca/main.php)^43^. The postmortem brain was paraffin-embedded, coronally sliced into 7,400 20-μm sections, silver-stained for cell bodies^90^ and digitized. Manual inspection for artefacts (that is, rips, tears, shears and stain crystallization) was followed by automatic repair procedures, involving nonlinear alignment to a postmortem MRI of the same individual acquired before sectioning, together with intensity normalization and block averaging^91^. The 3D reconstruction was implemented with a successive coarse-to-fine hierarchical procedure. We downloaded the 3D volume at 100-μm resolution, which was the highest resolution available for the whole brain. Computations were performed on inverted images, where intensity reflects greater cellular density and soma size. Geometric meshes approximating the outer and inner cortical interface (that is, the gray matter/cerebrospinal fluid boundary and the gray matter/white matter boundary) with 163,842 matched vertices per hemisphere were also obtained^92^.

We constructed 50 equivolumetric surfaces between the outer and inner cortical surfaces^93^. The equivolumetric model compensates for cortical folding by varying the Euclidean distance, *ρ*, between pairs of intracortical surfaces throughout the cortex to preserve the fractional volume between surfaces^94^; *ρ* was calculated as follows for each surface:

1 $$\rho =\frac{1}{{A}_{{\rm{out}}}-{A}_{{\rm{in}}}}\times\left(-{A}_{{\rm{in}}}+\sqrt{\alpha {A}_{{\rm{out}}}^{2}+\left(1-\alpha \right){A}_{{\rm{in}}}^{2}}\right)$$

where *α* represents fraction of the total volume of the segment accounted for by the surface, and *A*_out_ and *A*_in_ represent the surface area of the outer and inner cortical surfaces, respectively. Vertex-wise staining intensity profiles were generated by sampling cell-staining intensities along linked vertices from the outer to the inner surface. Smoothing was employed in tangential and axial directions to ameliorate the effects of artefacts, blood vessels and individual neuronal arrangement. The tangential smoothing across depths was enacted for each staining profile independently, using an iterative piece-wise linear procedure that minimizes shrinkage (three iterations^95^). Axial surface-wise smoothing was performed at each depth independently and involved moving a two-vertex full-width at half-maximum Gaussian kernel across the surface mesh using SurfStat in MATLAB^96,97^. The staining intensity profiles are available in the BigBrainWarp toolbox^44^.

### Comparison of cortical atlases

Functional networks were defined using a widely used atlas^1^. The atlas reflects clustering of cortical vertices according to similarity in resting-state functional connectivity profiles, acquired in 1,000 healthy young adults. Cortical types were assigned to Von Economo areas^42,98^, based on a recent reanalysis of Von Economo micrographs^24^. This classification scheme was used because its criteria are (1) clearly defined, (2) applied consistently across the entire cortex, (3) align with Von Economo’s original descriptions and (4) are supported by several histological samples. Criteria included ‘development of layer IV, prominence (denser cellularity and larger neurons) of deep (V–VI) or superficial (II–III) layers, definition of sublayers (for example, IIIa and IIIb), sharpness of boundaries between layers and presence of large pyramids in superficial layers’^24^. Thereby, cortical types synopsize degree of granularity, from high laminar elaboration in koniocortical areas, six identifiable layers in Eu-III to -I, poorly differentiated layers in dysgranular and absent layers in agranular.

The proportion of DMN vertices assigned to each cortical type was calculated on a common surface template, fsaverage5 (ref. ^99^). The equivalence of cortical type proportions in the DMN and each other functional network was evaluated via pairwise Kolgomorov–Smirnoff tests. Significant over- or under-representation of each cortical type within the DMN was evaluated with spin permutation testing^100^. Spin permutation testing, used throughout subsequent statistical analyses, involves generating a null distribution by rotating one brain map 10,000 times and recomputing the outcome of interest. We then calculate ${P}_{{\rm{spin}}}=1-\frac{\Sigma ({\rm{empirical}} > {\rm{permutations}})}{{\rm{total}\; \rm{permutations}}}$ and/or ${P}_{{\rm{spin}}}=1-\frac{\Sigma ({\rm{empirical}} < {\rm{permutations}})}{{\rm{total}\; \rm{permutations}}}$^101^. The null distribution preserves the spatial structure of both brain maps, which establishes the plausibility of a random alignment of the maps explaining their statistical correspondence. Generally, we deemed significance *P* < 0.05 for one-tailed tests and *P* < 0.025 for two-tailed tests. Additionally, we used Bonferroni correction when multiple univariate comparisons were made using the same response variable. In the case of over- or under-representation of specific cortical types within the DMN, we randomly rotated the cortical type atlas, then generated null distributions, representing the number of vertices within the DMN assigned to each type.

The robustness of cytoarchitectural heterogeneity to the DMN definition was assessed with three alternative atlases. Given the origins of the DMN as a task-negative set of regions^102^, the first alternative atlas involved identifying regions that are consistently deactivated during externally oriented tasks. In line with a recent review^15^, we used predefined contrast maps from 787 healthy young adults of the Human Connectome Project (HCP_S900_GroupAvg_v.1 Dataset). Each map represents the contrast between BOLD response during a task and at baseline. Fifteen tasks were selected to correspond to early studies of the DMN^103^ (working memory (WM)–2 back, WM-0 back, WM-body, WM-face, WM-place, WM-tool, gambling-punish, gambling-reward, motor-average, social-random, social-theory of mind, relational-match, relational-relation, emotion-faces, emotion-shapes). For each contrast, task-related deactivation was classed as *z* score ≤ −5, which is consistent with contemporary statistical thresholds used in neuroimaging to reduce false positives^104^. The second alternative atlas represented an independent component analysis of 7,342 task fMRI contrasts. The DMN was specified as the fourth component. The volumetric *z* statistic map for that component was projected to the cortical surface for analysis. Thirdly, a probabilistic atlas of the DMN was calculated as the percentage of contrasts with task-related deactivation. The second alternative atlas represented the probability of the DMN at each vertex, calculated across 1,029 individual-specific functional network delineations^46^. For each alternative atlas, we calculated the proportions of cortical types across a range of probabilistic thresholds (5–95%, at 5% increments) to determine whether the discovered cytoarchitectural heterogeneity of the DMN was robust to atlas definition.

### Data-driven cytoarchitectural axis within the DMN

The functional network atlas was transformed to the BigBrain surface using a specially optimized multimodal surface matching algorithm^44,48^. The pattern of cytoarchitectural heterogeneity in the DMN was revealed using nonlinear manifold learning. The approach involved calculating pairwise product–moment correlations of BigBrain staining intensity profiles, controlling for the average staining intensity profile within the DMN. Negative values were zeroed to emphasize nonshared similarities. Diffusion map embedding of the correlation matrix was employed to gain a low dimensional representation of cytoarchitectural patterns^49,100^. Diffusion map embedding belongs to the family of graph Laplacians, which involve constructing a reversible Markov chain on an affinity matrix. Compared with other nonlinear manifold learning techniques, the algorithm is relatively robust to noise and computationally inexpensive. A single parameter α controls the influence of the sampling density on the manifold (*α* = 0, maximal influence; *α* = 1, no influence). As in previous studies^19,25,100^, we set *α* = 0.5—a choice retaining the global relations between datapoints in the embedded space. Notably, different alpha parameters had little to no impact on the first eigenvector (spatial correlation of eigenvectors, *r* > 0.99).

The DMN comprised 71,576 vertices on the BigBrain surface, each associated with approximately 1 mm^2^ of surface area; however, pairwise correlation and manifold learning on 71,576 datapoints was computationally infeasible. Thus, we performed a sixfold mesh decimation on the BigBrain surface to select a subset of vertices that preserve the overall shape of the mesh. Then, we assigned each nonselected vertex to the nearest maintained vertex, determined by shortest path on the mesh (ties were solved by shortest Euclidean distance). Staining intensity profiles were averaged within each surface patch of the DMN, then the dimensionality reduction procedure was employed. Subsequent analyses focused on E1, which explained the most variance in the affinity matrix (approximately 28% of variance). Additionally, we repeated this analysis with a highly conservative delineation of the DMN (generated by using the intersection of the three abovementioned alternative atlases), thereby demonstrating that slight variations in atlas definition do not impact the organization of cytoarchitecture that we discovered in the network. To ensure the spatial pattern depicted by E1 was not purely a product of the selected dimensionality reduction method, we also repeated the procedure using principal component analysis and Laplacian eigenmaps. The first components were near-identical across all approaches (*r* > 0.99).

Local variations in E1 were examined within spatially contiguous subregions of the DMN. Subregions were defined programmatically on the cortical mesh, named according to the gyri they primarily occupy and compared with the Von Economo parcellation (Von Economo areas occupying >10% of the subregion are listed in ascending order in the following): superior frontal and anterior cingulate cortex (FCBm, FB, FA, FDT), middle temporal (TD, PH), inferior parietal (PF, PD, TD), precuneus (PD, LA2, LC1), inferior frontal (FE FDdelta) and parahippocampal (HB). Quantitative description of E1 topography within each subregion was achieved with two complementary approaches. First, to characterize the smoothness and complexity of the landscape, we fit polynomial models between E1 and two spatial axes^105^. The spatial axes were derived from an Isomax flattening of each subregion, resulting in a two-dimensional (2D) description of each subregion. We compared adjusted *R*^2^ between subregions within each polynomial order (quadratic, cubic and quartic) using a one-way analysis of variance, whereby each subregion was represented by a left and right hemisphere observation. Second, to characterize the bumpiness of subregion landscapes, we adopted an approach from material engineering for characterizing the roughness of a surface^51,106^. Specifically, we calculated a waviness metric that reflects the number of intersections of the zero-plane while accounting for the size of the region. As above, we compared waviness between subregions using a one-way analysis of variance. Notably, the sensitivity of each approach to variations in E1 topography was validated in a series of simulations, in which we modulated the flatness and bumpiness of the input landscape (Extended Data Fig. 5).

### MRI acquisition and processing—primary analyses

Primary MRI analyses were conducted on 40 healthy adults from the microstructure informed connectomics cohort (14 female, mean ± s.d. age, 30.4 ± 6.7 years, two left-handed)^107^. Scans were completed at the Brain Imaging Center of the Montreal Neurological Institute and Hospital on a 3-T Siemens Magnetom Prisma-Fit equipped with a 64-channel head coil. Two T1w scans with identical parameters were acquired with a 3D-MPRAGE sequence (0.8-mm isotropic voxels, TR = 2,300 ms, TE = 3.14 ms, TI = 900 ms, flip angle = 9°, iPAT = 2, matrix = 320 × 320, 224 sagittal slices, partial Fourier = 6/8). T1w scans were inspected visually to ensure minimal head motion before they were submitted to further processing. A spin-echo echo-planar imaging sequence with multiband acceleration was used to obtain diffusion-weighted imaging (DWI) data, consisting of three shells with *b* values of 300, 700 and 2,000 s mm^−2^ and 10, 40 and 90 diffusion weighting directions per shell, respectively (1.6-mm isotropic voxels, TR = 3,500 ms, TE = 64.40 ms, flip angle = 90°, refocusing flip angle = 180°, FOV = 224 × 224 mm^2^, slice thickness =1.6 mm, multiband factor = 3, echo spacing = 0.76 ms, number of b0 images = 3). One 7-min rs-fMRI scan was acquired using multiband accelerated 2D-BOLD echo-planar imaging (3-mm isotropic voxels, TR = 600 ms, TE = 30 ms, flip angle = 52°, FOV = 240 × 240 mm^2^, slice thickness = 3 mm, multiband factor = 6, echo spacing = 0.54 ms). Participants were instructed to keep their eyes open, look at a fixation cross and not fall asleep. Two spin-echo images with reverse-phase encoding were also acquired for distortion correction of the rs-fMRI scans (phase encoding = AP/PA, 3-mm isotropic voxels, FOV = 240 × 240 mm^2^, slice thickness = 3 mm, TR = 4,029 ms, TE = 48 ms, flip angle = 90°, echo spacing = 0.54 ms, bandwidth = 2,084 Hz per pixel).

An open-access tool was used for multimodal data processing^108^. Each T1w scan was deobliqued and reoriented. Both scans were then linearly coregistered and averaged, automatically corrected for intensity nonuniformity^109^ and intensity normalized. Resulting images were skull-stripped, and nonisocortical structures were segmented using FSL FIRST^110^. Different tissue types (cortical and subcortical gray matter, white matter, cerebrospinal fluid) were segmented to perform anatomically constrained tractography^111^. Cortical surface segmentations were generated from native T1w scans using FreeSurfer v.6.0 (refs. ^99,112,113^). DWI data were preprocessed using MRtrix^114,115^. DWI data underwent b0 intensity normalization, and were corrected for susceptibility distortion, head motion and eddy currents. Required anatomical features for tractography processing (for example, tissue type segmentations, parcellations) were nonlinearly coregistered to native DWI space using the deformable SyN approach implemented in Advanced Neuroimaging Tools (ANTs)^116^. Diffusion processing and tractography were performed in native DWI space. We performed anatomically constrained tractography using tissue types segmented from each participant’s preprocessed T1w images registered to native DWI space^111^. We estimated multishell and multitissue response functions^117^ and performed constrained spherical deconvolution and intensity normalization^118^. We initiated the tractogram with 40 million streamlines (maximum tract length, 250; fractional anisotropy cutoff, 0.06). We applied spherical deconvolution informed filtering of tractograms (SIFT2) to reconstruct whole-brain streamlines weighted by cross-sectional multipliers^119^. The reconstructed cross-section streamlines were averaged within 400 spatially contiguous, functionally defined parcels^120^, also warped to DWI space. The rs-fMRI images were preprocessed using AFNI^121^ and FSL^110^. The first five volumes were discarded to ensure magnetic field saturation. Images were reoriented, motion corrected and distortion corrected. Nuisance variable signal was removed using an ICA-FIX classifier^122^ and by performing spike regression. Native timeseries were mapped to individual surface models using a boundary-based registration^123^ and smoothed using a Gaussian kernel (full-width at half-maximum = 10 mm, smoothing performed on native midsurface mesh) using workbench^124^. For isocortical regions, timeseries were sampled on native surfaces and averaged within 400 spatially contiguous, functionally defined parcels^120^. For nonisocortical regions, timeseries were averaged within native parcellations of the nucleus accumbens, amygdala, caudate nucleus, hippocampus, pallidum, putamen and thalamus^110^.

### MRI acquisition and processing—secondary analyses

Secondary MRI analyses were conducted in 100 unrelated healthy adults (66 female, mean ± s.d. age = 28.8 ± 3.8 years) from the minimally preprocessed S900 release of the Human Connectome Project (HCP)^124,125^. MRI data were acquired on the HCP’s custom 3-T Siemens Skyra equipped with a 32-channel head coil. Two T1w images with identical parameters were acquired using a 3D-MPRAGE sequence (0.7-mm isotropic voxels, TE = 2.14 ms, TI = 1,000 ms, flip angle = 8°, iPAT = 2, matrix = 320 × 320, 256 sagittal slices; TR = 2,400 ms). Two T2w images were acquired using a 3D T2-SPACE sequence with identical geometry (TR = 3,200 ms, TE = 565 ms, variable flip angle, iPAT = 2). A spin-echo echo-planar imaging sequence was used to obtain diffusion-weighted images, consisting of three shells with *b* values 1,000; 2,000 and 3,000 s mm^−2^ and up to 90 diffusion weighting directions per shell (TR = 5,520 ms, TE = 89.5 ms, flip angle = 78°, refocusing flip angle = 160°, FOV = 210 × 180, matrix = 178 × 144, slice thickness = 1.25 mm, mb factor = 3, echo spacing = 0.78 ms). Four rs-fMRI scans were acquired using multiband accelerated 2D-BOLD echo-planar imaging (2-mm isotropic voxels, TR = 720 ms, TE = 33 ms, flip angle = 52°, matrix = 104 × 90, 72 sagittal slices, multiband factor = 8, 1,200 volumes per scan, 3,456 s). Only the first session was investigated in the present study. Participants were instructed to keep their eyes open, look at a fixation cross and not fall asleep. Nevertheless, some subjects were drowsy and may have fallen asleep^126^, and the group-averages investigated in the present study do not address these interindividual differences.

MRI data underwent HCP’s minimal preprocessing^124^. Cortical surface models were constructed using Freesurfer v.5.3-HCP^99,112,113^, with minor modifications to incorporate both T1w and T2w^127^. Diffusion MRI data underwent correction for geometric distortions and head motion^124^. Tractographic analysis was based on MRtrix3 (refs. ^114,115^). Response functions for each tissue type were estimated using the dhollander algorithm^128^. Fiber orientation distributions (that is, the apparent density of fibers as a function of orientation) were modeled from the diffusion-weighted MRI with multishell multitissue spherical deconvolution^118^, then values were normalized in the log domain to optimize the sum of all tissue compartments toward 1, under constraints of spatial smoothness. Anatomically constrained tractography was performed systematically by generating streamlines using second-order integration over fiber orientation distributions with dynamic seeding^119,129^. Streamline generation was aborted when 40 million streamlines had been accepted. We applied spherical deconvolution informed filtering of tractograms (SIFT2) to reconstruct whole-brain streamlines weighted by cross-sectional multipliers. The reconstructed cross-section streamlines were averaged within 400 spatially contiguous, functionally defined parcels^120^, also warped to DWI space. The rs-fMRI timeseries were corrected for gradient nonlinearity, head motion, bias field and scanner drifts, then structured noise components were removed using ICA-FIX, further reducing the influence of motion, non-neuronal physiology, scanner artefacts and other nuisance sources^122^. The rs-fMRI data were resampled from volume to MSMAll functionally aligned surface space^130,131^ and averaged within 400 spatially contiguous, functionally defined parcels^120^.

### Modeling structural connectivity with *E*_nav_

Connectivity of DMN subunits was mapped using structural connectomes, derived from diffusion-based tractography. Edge weights of the structural connectomes (*W*), representing number of streamlines, were remapped using a log-based transformation: (−log_10_(*W*/(max(*W*) + min(*W* > 0))). This log-based transformation attenuates extreme weights and ensures the maximum edge weight is mapped to a positive value. Euclidean distances were calculated between the centroid coordinate of each parcel. Communication in the structural connectome was modeled using navigation^56^, also known as greedy routing^132^. Navigation combines the structural connectome with physical distances, providing a routing strategy that recapitulates invasive, tract-tracing measures of communication^39^. In brief, navigation involves identifying a single, efficient path between two nodes, where each step is determined by spatial proximity to target node. Specifically, the next node in the path is the neighbor of the current node (that is, sharing a structural connection) that is closest to the final target node. Navigation is the sum distances of the selected path and *E*_nav_ its inverse; providing an intuitive metric of communication efficiency between two regions. *E*_nav_ was calculated within each hemisphere separately, then concatenated for analyses.

By integrating both topological as well as geometric information in the routing strategy, navigation achieves a topological balance between regularity and randomness that is common for small-world networks such as the human brain^133^. Thus, the approach addresses distance bias in group-representative structural connectomes^134^. In previous evaluations^39,56^, navigation was found to both promote a resource-efficient distribution of network information traffic and to explain variation in resting-state functional connectivity. Unlike other commonly studied communication strategies in connectomics (for example, shortest path routing), navigation does not involve global knowledge of network topology during the node-to-node propagation but simply follows a greedy routing strategy that can be implemented locally, supporting its biological plausibility.

### Modeling functional input and output with effective connectivity

The position of the DMN in large-scale cortical dynamics was explored with rDCM^40^—a scalable generative model of effective connectivity that allows inferences on the directionality of signal flow, openly available as part of the TAPAS software package^135^. Effective connectivity aims to describe directed interactions among brain regions, with estimates describing how different regions influence each other’s timeseries. Typically, effective connectivity parameters are estimated in a Bayesian framework by solving a set of differential equations in the time domain (that is, classic DCM), but computational cost of model inversion limits the number of regions that can be included. rDCM overcomes this limitation by converting the equations into an efficiently solvable Bayesian linear regression in the frequency domain. In doing so, rDCM allows computation of effective connectivity parameters for hundreds of brain regions. In previous work, the face and construct validity of rDCM for inferring effective connectivity parameters during resting state has been established using comprehensive simulations and by comparing rDCM against alternative generative models of rs‐fMRI data for small networks^136^.

The rDCM was implemented using individual rs-fMRI timeseries. Additionally, an extended version of the rDCM was generated with nonisocortical regions, specifically the nucleus accumbens, amygdala, caudate nucleus, hippocampus, pallidum, putamen and thalamus.

### Influence of cytoarchitecture on connectivity

Each parcel was labeled according to functional network, modal cortical type and, if part of the DMN, average E1 value. Parcel-average E1 values were calculated by transforming the parcellation scheme to the BigBrain surface and averaging within each parcel^44,48^. The following analyses were repeated for *E*_nav_, effective connectivity derived input and effective connectivity derived output.

First, we selected DMN rows and non-DMN columns of the connectivity matrix. Then, we performed product–moment correlations between E1 and average connectivity to assess the association of the cytoarchitectural axis with connectivity. Next, we stratified the non-DMN columns by cortical type, averaged within type and calculated product–moment correlation between type-average connectivity and E1, providing more specific insight into the relation of the cytoarchitectural axis with connectivity of certain cortical types. For each modality, the correlations were compared with 10,000 spin permutations. *P* values were Bonferroni corrected for seven comparisons, resulting in significance threshold of *P* < 0.004 (two-sided test with alpha value of 0.05).

Finally, we estimated the imbalance in connectivity to each cortical type by calculating average connectivity to each type, then calculating the Kullback–Leibler (KL) divergence from a null model with equal average connectivity to each type. The imbalance analysis was repeated for each functional network. In each case, only internetwork connections were included in the calculations. For each modality and each network, we tested whether the KL divergence value was lower than 10,000 spin permutations. *P* values were Bonferroni corrected for seven comparisons, resulting in significance threshold of *P* < 0.007 (one-sided test with alpha value of 0.05).

### Individual-level replication with high-field MRI

In the replication, we sought to address two key limitations of the primary analyses. First, due to the unique nature of the BigBrain dataset, cytoarchitectural mapping was based on a single person, limiting our knowledge of the generalizability of the discovered patterns. Second, structural and functional connectivity measurements represented population averages, thus we were not able to conclude whether the discovered correspondences between cytoarchitecture and connectivity are evident within an individual. To overcome these limitations, we sought to replicate key findings at an individual level using high-resolution, ultrahigh-field MRI.

Individual-level replication analyses were conducted on eight healthy adults (five female, mean ± s.d. age = 28 ± 6.3, one left-handed). The MRI data acquisition protocols were approved by the Research Ethics Board of McGill University. All participants provided written informed consent, which included a provision for openly sharing all data in anonymized form. Scans were completed at the Brain Imaging Center of the Montreal Neurological Institute and Hospital on a 7-T Siemens Magnetom Terra System equipped with a 32/8 channel receive/transmit head coil. Two qT1 scans were acquired across two scanning sessions with identical 3D-MP2RAGE sequences (0.5-mm isotropic voxels, TR = 5,170 ms, TE = 2.44 ms, T1_1/2_ = 1,000/3,200 ms, flip angles = 4°, matrix = 488 × 488, slice thickness = 0.5 mm, partial Fourier = 0.75). qT1 maps from the second session were registered linearly to the qT1 maps from the first session, then averaged to enhanced the signal to noise ratio. A spin-echo echo-planar imaging sequence with multiband acceleration was used to obtain DWI data, consisting of three shells with *b* values 300, 700 and 2,000s mm^−2^ and 10, 40 and 90 diffusion weighting directions per shell, respectively (1.1-mm isotropic voxels, TR = 7,383 ms, TE = 70.6 ms, flip angle = 90°, matrix = 192 × 192, slice thickness = 1.1 mm, multiband factor = 2, echo spacing = 0.26 ms, number of b0 images = 3, partial Fourier = 0.75). One 6-min rs-fMRI scan was acquired using multi-echo, multiband accelerated 2D-BOLD echo-planar imaging (1.9-mm isotropic voxels, TR = 1,690 ms, TE_1/2/3_ = 10.8/27.3/43.8 ms, flip angle = 67°, matrix = 118 × 118, multiband factor = 3, echo spacing = 0.54 ms, partial Fourier = 0.75). Participants were instructed to keep their eyes open, look at a fixation cross and not fall asleep. Two multiband accelerated spin-echo images with reverse-phase encoding were also acquired for distortion correction of the rs-fMRI scans.

The 7 T dataset was processed in the same manner as the primary MRI dataset, with two exceptions. qT1 maps were used, rather than T1w images, to construct cortical surfaces, and nuisance variable signal was removed from rs-fMRI using an approach that is specially tailored to multi-echo fMRI (tedana)^137^, instead of ICA-FIX, which is optimized for single-echo data. Subsequently, we extracted intracortical profiles from qT1 volumes and determined E1 of microstructural differentiation for each individual using the same procedure as for the histological data. In addition, we used the preprocessed resting-state timeseries to produce individual-specific parcellations for each subject, via a pretrained hierarchical Bayesian model^138^. We subsequently used these parcellations to obtain individual-specific DMNs.

The replication focused on three key results from the primary analysis: (1) DMN subregions differ in terms of the topography of microarchitectural differentiation, which is evident in the roughness of E1. In particular, subregions vary from a gradient in the mesiotemporal lobe to a fluctuating landscape in the prefrontal cortex. (2) *E*_nav_ decreases along E1, and this effect is especially pronounced for perceptually coupled cortical types (koniocortical and Eu-III). (3) Functional input decreases along E1. For each result, we compared statistical outcomes of the primary analysis, derived from BigBrain and population-average connectivity, with individual-level statistical outcomes, derived from the 7-T dataset, using product–moment correlations. We report rho and *P* values averaged across individual participants.

### Reporting summary

Further information on research design is available in the Nature Portfolio Reporting Summary linked to this article.

## Online content

Any methods, additional references, Nature Portfolio reporting summaries, source data, extended data, supplementary information, acknowledgements, peer review information; details of author contributions and competing interests; and statements of data and code availability are available at 10.1038/s41593-024-01868-0.

## Supplementary information

Supplementary Information Supplementary Methods, Fig. 1 and Tables 1–3.

Reporting Summary

## Figures and Tables

**Extended Data Table 1:**
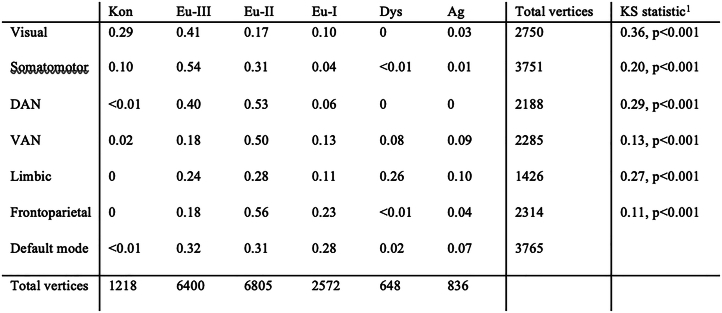
Cortical types by functional network

^1^Two-sided Kolmogorov-Smirnov tests for independence of samples were calculated between each network and the DMN.

*Note:* entries in the centre of the table are proportions, which are provided relative to the functional network (ie: 29% of the visual network is koniocortical), thereby the rows approximately sum to 1 (given rounding errors).

Kon=koniocortical. Eu=eulaminate. Dys=dysgranular. Ag=agranular. DAN=dorsal attention network. VAN=ventral attention network.

## Data Availability

All data that support the findings of this study are openly available. BigBrain is available with LORIS (https://bigbrain.loris.ca/main.php)^55^ with preprocessed BigBrain data available in through the BigBrainWarp GitHub repository (https://github.com/caseypaquola/BigBrainWarp)^56^. The MICS dataset is available with CONP Portal (https://portal.conp.ca/dataset?id=projects/mica-mics)^130^ and the HCP dataset is available with Connectome DB (https://db.humanconnectome.org/)^124^.

## Code availability

Custom MATLAB (v.2022b)^96^ code for this study, as well as data necessary for reproduction, are openly available via GitHub at https://github.com/caseypaquola/DMN and Zenodo at 10.5281/zenodo.14034720 (ref. ^139^).
