# Author Correction: The architecture of the human default mode network explored through cytoarchitecture, wiring and signal flow

**DOI:** 10.1038/s41593-025-01900-x

**Published:** 2025-02-11

**Authors:** Casey Paquola, Margaret Garber, Stefan Frässle, Jessica Royer, Yigu Zhou, Shahin Tavakol, Raul Rodriguez-Cruces, Donna Gift Cabalo, Sofie Valk, Simon B. Eickhoff, Daniel S. Margulies, Alan Evans, Katrin Amunts, Elizabeth Jefferies, Jonathan Smallwood, Boris C. Bernhardt

**Affiliations:** 1 McConnell Brain Imaging Centre, Montreal Neurological Institute, McGill University, Montréal, Quebec Canada; 2 Institute for Neuroscience and Medicine (INM-7), Forschungszentrum Jülich, Jülich, Germany; 3 Translational Neuromodeling Unit (TNU), University of Zurich and ETH Zurich, Zurich, Switzerland; 4 Max Planck Institute for Cognitive and Brain Sciences, Leipzig, Germany; 5 Institute for Systems Neuroscience, Heinrich Heine Universistät Dusseldorf, Dusseldorf, Germany; 6 Integrative Neuroscience & Cognition Center (INCC – UMR 8002), University of Paris, Centre national de la recherche scientifique (CNRS), Paris, France; 7 Institute for Neuroscience and Medicine (INM-1), Forschungszentrum Jülich, Jülich, Germany; 8 Department of Psychology, University of York, York, UK; 9 Department of Psychology, Queen’s University, Kingston, Ontario Canada

**Keywords:** Cognitive neuroscience, Computational neuroscience

Correction to: *Nature Neuroscience* 10.1038/s41593-024-01868-0, published online 28 January 2025.

In the version of the article initially published, the initial “B.” was missing from Simon B. Eickhoff’s name and has now been added to the HTML and PDF versions of the article.

