# Supplementary material for: The architecture of the human default mode network explored through cytoarchitecture, wiring and signal flow
Source: Nat Neurosci. 2025 Jan 28;28(3):654–64. doi: 10.1038/s41593-024-01868-0 (PMC11893468; doi:10.1038/s41593-024-01868-0)
Supplement: Supplementary file 1 — Supplementary Methods, Fig. 1 and Tables 1–3. [file 41593_2024_1868_MOESM1_ESM.pdf]

nature neuroscience

Supplementary information

https://doi.org/10.1038/s41593-024-01868-0

The architecture of the human default mode network explored through cytoarchitecture, wiring and signal flow

In the format provided by the authors and unedited

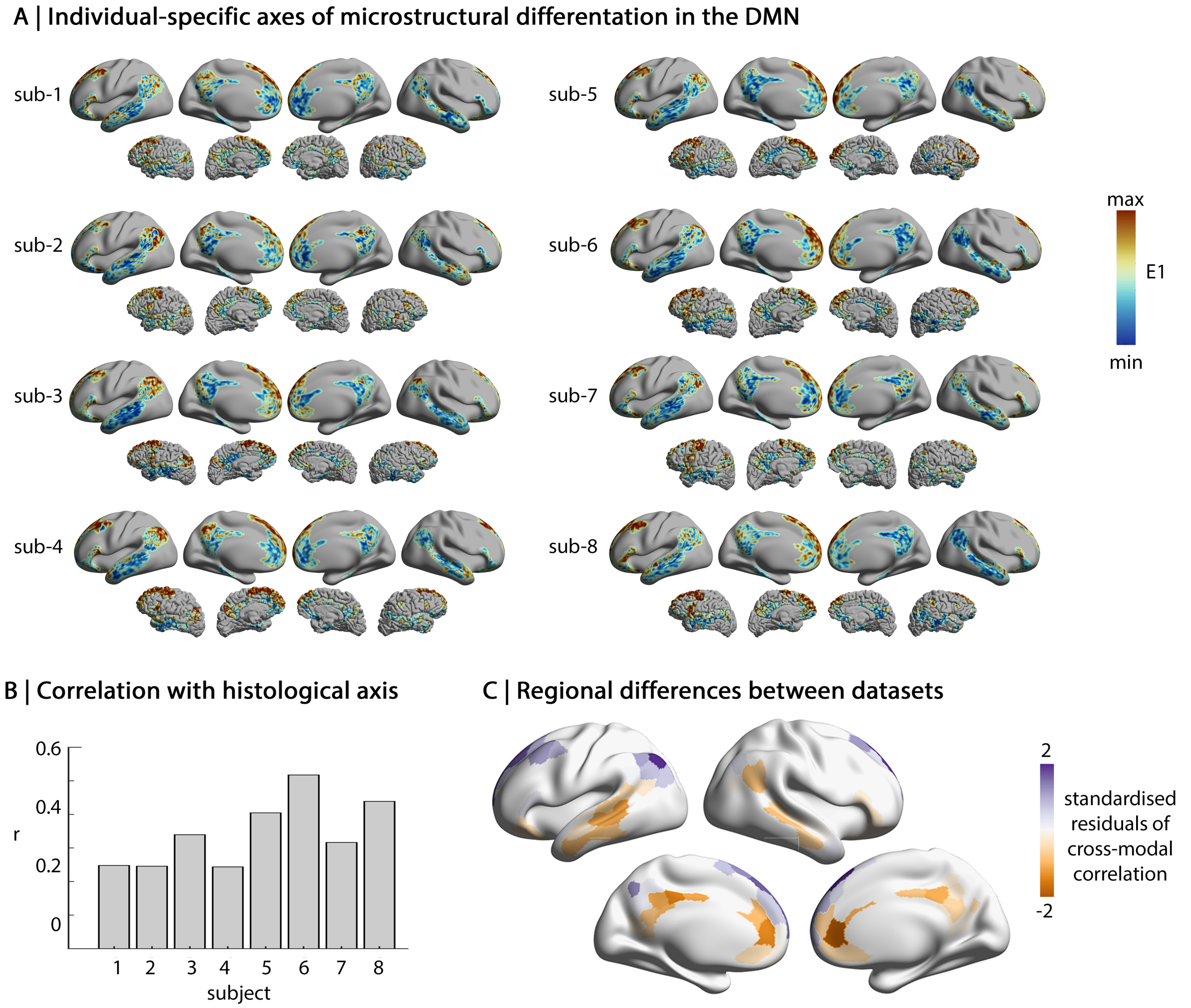

Supplementary Figure 1: Microstructural axes derived from 7T MRI. A) The principal eigenvector of microstructural variation in the DMN (E1) extracted from myelin-sensitive quantitative MRI (qT1) is displayed for all eight subjects. For each subject, E1 is presented using atlas-based DMN on a standard surface (above) and individual-specific DMN on the subject's cortical surface (below). Notably, E1 was very similar between atlas- and individual-based reconstructions of the DMN. **B)** Subject-specific correlations of the qT1-derived axis with the axis of histological variation derived from BigBrain. **C)** Group-average standardised residuals highlight which parcels deviate from cross-modal alignment. Residuals were calculated by fitting a linear model between each subject-specific axis and the histological axis. Dark purple in the left lateral parietal area signifies higher E1 in the MRI dataset relative to the histological dataset, whereas dark orange in the anterior cingulate indicates lower E1 in the MRI dataset.

Supplementary Table 1: Parcel-wise characteristics

E1	Parcel name	Subregion	Von Economo	Cortical type	Enav	Afferent
-4.01	7Networks_LH_Default_Par_3	Inferior parietal	PG	Eu-I	2.0E-02	7.0E-04
-3.80	7Networks_LH_Default_pCunPCC_1	Precuneus	LC2	Eu-I	2.0E-02	7.3E-04
-3.70	7Networks_LH_Default_pCunPCC_10	Precuneus	PE	Eu-III	2.0E-02	9.0E-04
-3.63	7Networks_LH_Default_Par_5	Inferior parietal	PG	Eu-I	2.2E-02	5.6E-04
-3.62	7Networks_RH_Default_pCunPCC_2	Precuneus	LE	Agranular	2.0E-02	6.9E-04
-3.59	7Networks_RH_Default_pCunPCC_1	Precuneus	OA	Eu-III	2.1E-02	7.8E-04
-3.53	7Networks_LH_Default_Par_6	Inferior parietal	PG	Eu-I	2.0E-02	7.5E-04
-3.25	7Networks_RH_Default_pCunPCC_5	Precuneus	PE	Eu-III	2.0E-02	7.7E-04
-3.14	7Networks_LH_Default_Temp_4	Middle temporal	TE	Eu-III	1.9E-02	4.6E-04
-3.09	7Networks_LH_Default_Temp_6	Middle temporal	TE	Eu-III	2.0E-02	6.0E-04
-3.06	7Networks_RH_Default_Par_2	Inferior parietal	PG	Eu-I	2.1E-02	7.3E-04
-2.94	7Networks_LH_Default_Temp_2	Middle temporal	TE	Eu-III	1.7E-02	4.7E-04
-2.89	7Networks_RH_Default_Temp_7	Middle temporal	TE	Eu-III	2.0E-02	6.9E-04
-2.79	7Networks_RH_Default_Temp_2	Middle temporal	TE	Eu-III	1.6E-02	4.4E-04
-2.74	7Networks_RH_Default_Temp_1	Middle temporal	TG	Dysgranular	1.5E-02	3.4E-04
-2.58	7Networks_RH_Default_pCunPCC_4	Precuneus	LC1	Eu-I	2.0E-02	7.1E-04
-2.53	7Networks_LH_Default_pCunPCC_6	Precuneus	PE	Eu-III	2.0E-02	6.8E-04
-2.48	7Networks_LH_Default_Temp_7	Middle temporal	TE	Eu-III	2.1E-02	5.6E-04
-2.41	7Networks_LH_Default_Temp_3	Middle temporal	TE	Eu-III	1.8E-02	5.2E-04
-2.40	7Networks_LH_Default_Par_7	Inferior parietal	PG	Eu-I	2.1E-02	6.8E-04
-2.36	7Networks_RH_Default_Temp_4	Middle temporal	TE	Eu-III	1.9E-02	5.0E-04
-2.31	7Networks_LH_Default_Temp_10	Middle temporal	TE	Eu-III	2.2E-02	6.2E-04
-2.25	7Networks_LH_Default_pCunPCC_11	Precuneus	PE	Eu-III	2.1E-02	6.8E-04
-2.15	7Networks_RH_Default_Par_1	Inferior parietal	TE	Eu-III	2.3E-02	6.6E-04
-2.06	7Networks_LH_Default_Par_1	Inferior parietal	TE	Eu-III	2.2E-02	6.3E-04
-1.91	7Networks_LH_Default_pCunPCC_4	Precuneus	LE	Agranular	2.0E-02	7.4E-04
-1.84	7Networks_RH_Default_Par_5	Inferior parietal	PG	Eu-I	2.1E-02	7.9E-04
-1.81	7Networks_LH_Default_pCunPCC_2	Precuneus	PE	Eu-III	2.2E-02	7.2E-04
-1.70	7Networks_RH_Default_Temp_6	Middle temporal	TE	Eu-III	2.1E-02	6.2E-04
-1.66	7Networks_LH_Default_Temp_1	Middle temporal	TE	Eu-III	1.6E-02	3.6E-04
-1.58	7Networks_RH_Default_Temp_3	Middle temporal	TG	Dysgranular	1.7E-02	4.4E-04
-1.05	7Networks_LH_Default_pCunPCC_3	Precuneus	LC1	Eu-I	1.9E-02	7.1E-04
-0.95	7Networks_RH_Default_Par_3	Inferior parietal	PG	Eu-I	2.2E-02	5.9E-04
-0.95	7Networks_RH_Default_Temp_8	Middle temporal	TE	Eu-III	2.2E-02	6.9E-04
-0.94	7Networks_LH_Default_Par_4	Inferior parietal	PG	Eu-I	2.2E-02	6.5E-04
-0.76	7Networks_LH_Default_Par_2	Inferior parietal	PG	Eu-I	2.2E-02	5.9E-04
-0.74	7Networks_LH_Default_PFC_15	Superior frontal and ACC	FD	Eu-II	1.5E-02	5.4E-04
-0.64	7Networks_LH_Default_PFC_10	Inferior frontal	FCBm	Eu-II	1.9E-02	5.4E-04
-0.53	7Networks_LH_Default_PFC_19	Superior frontal and ACC	FC	Eu-I	1.5E-02	3.8E-04
-0.52	7Networks_LH_Default_PFC_16	Superior frontal and ACC	FC	Eu-I	1.7E-02	4.8E-04
-0.46	7Networks_RH_Default_pCunPCC_8	Precuneus	PE	Eu-III	2.2E-02	6.4E-04
-0.37	7Networks_LH_Default_PFC_11	Superior frontal and ACC	FD	Eu-II	1.2E-02	4.5E-04
-0.30	7Networks_LH_Default_Temp_5	Middle temporal	TA	Eu-II	1.9E-02	5.9E-04
-0.08	7Networks_RH_Default_Par_4	Inferior parietal	PF	Eu-II	2.3E-02	7.6E-04
-0.05	7Networks_RH_Default_PFCdPFCm_6	Superior frontal and ACC	LA2	Agranular	1.6E-02	4.3E-04
0.08	7Networks_RH_Default_PFCdPFCm_5	Superior frontal and ACC	FD	Eu-II	1.2E-02	4.2E-04
0.11	7Networks_RH_Limbic_TempPole_7	Parahippocampal	HC	Agranular	1.7E-02	3.8E-04
0.11	7Networks_LH_Default_pCunPCC_7	Precuneus	LC1	Eu-I	2.1E-02	6.4E-04
0.28	7Networks_RH_Default_PFCv_3	Inferior frontal	FF	Eu-II	1.6E-02	5.5E-04
0.52	7Networks_RH_Default_PFCdPFCm_8	Superior frontal and ACC	FD	Eu-II	1.4E-02	3.8E-04
0.67	7Networks_RH_Default_Temp_5	Middle temporal	TE	Eu-III	1.9E-02	5.9E-04
0.69	7Networks_LH_Default_PFC_7	Inferior frontal	FDT	Eu-III	1.8E-02	6.0E-04
0.73	7Networks_LH_Default_PFC_20	Superior frontal and ACC	FB	Eu-II	2.1E-02	6.6E-04
0.77	7Networks_LH_Default_PFC_21	Superior frontal and ACC	FB	Eu-II	1.8E-02	5.3E-04
0.83	7Networks_RH_Default_PFCdPFCm_12	Superior frontal and ACC	FB	Eu-II	1.8E-02	4.5E-04
0.94	7Networks_LH_Default_PFC_22	Superior frontal and ACC	FB	Eu-II	1.8E-02	4.4E-04
0.96	7Networks_RH_Default_PFCv_1	Inferior frontal	FF	Eu-II	1.7E-02	4.0E-04
1.13	7Networks_LH_Default_PFC_6	Superior frontal and ACC	FD	Eu-II	1.4E-02	5.5E-04
1.16	7Networks_RH_Default_pCunPCC_7	Precuneus	LA1	Agranular	2.1E-02	7.4E-04
1.18	7Networks_RH_Default_PFCdPFCm_13	Superior frontal and ACC	FB	Eu-II	1.6E-02	4.6E-04
1.27	7Networks_RH_Default_PFCdPFCm_1	Superior frontal and ACC	FE	Eu-III	1.4E-02	4.5E-04
1.31	7Networks_RH_Default_PFCdPFCm_10	Superior frontal and ACC	FC	Eu-I	1.7E-02	4.5E-04
1.40	7Networks_RH_Default_pCunPCC_6	Precuneus	LC1	Eu-I	2.1E-02	6.5E-04
1.48	7Networks_LH_Default_PFC_17	Superior frontal and ACC	FB	Eu-II	1.8E-02	5.7E-04
1.48	7Networks_RH_Default_PFCv_4	Inferior frontal	FDT	Eu-III	1.9E-02	5.6E-04
1.49	7Networks_RH_Default_PFCdPFCm_2	Superior frontal and ACC	FD	Eu-II	1.1E-02	3.6E-04
1.50	7Networks_LH_Default_PFC_24	Superior frontal and ACC	FB	Eu-II	1.6E-02	4.6E-04

1.64	7Networks_LH_Default_PFC_4	Superior frontal and ACC	FH	Eu-II	1.4E-02	4.7E-04
1.68	7Networks_RH_Default_PFCdPFCm_11	Superior frontal and ACC	FC	Eu-I	1.4E-02	3.6E-04
1.69	7Networks_LH_Default_PFC_12	Superior frontal and ACC	LA1	Agranular	1.5E-02	4.3E-04
1.73	7Networks_LH_Default_PFC_5	Inferior frontal	FF	Eu-II	1.6E-02	5.8E-04
1.77	7Networks_LH_Default_PFC_3	Superior frontal and ACC	FE	Eu-III	1.2E-02	4.9E-04
1.82	7Networks_LH_Default_PFC_2	Inferior frontal	FF	Eu-II	1.6E-02	5.0E-04
1.83	7Networks_LH_Default_PFC_14	Superior frontal and ACC	FD	Eu-II	1.3E-02	4.4E-04
1.91	7Networks_RH_Default_PFCdPFCm_9	Superior frontal and ACC	FC	Eu-I	1.4E-02	3.8E-04
2.03	7Networks_LH_Default_pCunPCC_9	Precuneus	LA1	Agranular	2.0E-02	7.1E-04
2.05	7Networks_RH_Default_PFCv_2	Inferior frontal	FF	Eu-II	1.6E-02	4.3E-04
2.09	7Networks_LH_Default_PFC_18	Superior frontal and ACC	FC	Eu-I	1.3E-02	3.8E-04
2.18	7Networks_LH_Default_PFC_1	Inferior frontal	FF	Eu-II	1.8E-02	5.0E-04
2.24	7Networks_LH_Default_PFC_8	Superior frontal and ACC	FD	Eu-II	1.2E-02	4.9E-04
2.29	7Networks_LH_Limbic_TempPole_8	Parahippocampal	HC	Agranular	1.7E-02	3.7E-04
2.58	7Networks_LH_Default_pCunPCC_8	Precuneus	LC2	Eu-I	2.1E-02	6.7E-04
2.62	7Networks_RH_Default_PFCdPFCm_3	Superior frontal and ACC	LA1	Agranular	1.4E-02	4.3E-04
2.77	7Networks_LH_Default_PFC_13	Superior frontal and ACC	FD	Eu-II	1.2E-02	4.2E-04
3.15	7Networks_RH_Default_PFCdPFCm_4	Superior frontal and ACC	FD	Eu-II	1.3E-02	4.2E-04
3.29	7Networks_LH_Default_PFC_9	Superior frontal and ACC	LA1	Agranular	1.3E-02	4.6E-04
3.53	7Networks_RH_Default_PFCdPFCm_7	Superior frontal and ACC	FD	Eu-II	1.2E-02	3.9E-04
3.68	7Networks_LH_Default_PFC_23	Superior frontal and ACC	FB	Eu-II	1.5E-02	3.7E-04

Supplementary Table 2: Correlation of DMN connectivity with cytoarchitectural axis

Measure of connectivity	Dataset	All non-DMN	Koniocortical	Eulaminate-III	Eulaminate-II	Eulaminate-I	Dysgranular	Agranular
E_{NAV} (Structural model)	MICS	**r=-0.60, p<0.001**	**r=-0.63, p<0.001**	**r=-0.60, p<0.001**	r=-0.38, p=0.006	r=-0.26, p=0.094	r=0.09, p=0.291	r=0.26, p=0.051
	HCP	**r=-0.59, p<0.001**	**r=-0.43, p<0.001**	**r=-0.64, p<0.001**	r=-0.24, p=0.134	r=-0.14, p=0.232	r=0.08, p=0.328	r=0.34, p=0.038
Input (Functional model)	MICS	**r=-0.54, p<0.001**	**r=-0.38, p=0.006**	**r=-0.62, p<0.001**	**r=-0.35, p=0.005**	**r=-0.48, p=0.001**	r=-0.30, p=0.014	**r=-0.43, p=0.003**
	HCP	**r=-0.40, p<0.001**	r=-0.23, p=0.085	**r=-0.49, p<0.001**	**r=-0.33, p=0.003**	r=-0.20, p=0.044	r=-0.21, p=0.015	r=-0.18, p=0.140
Output (Functional model)	MICS	r=-0.18, p=0.064	r=-0.10, p=0.201	r=-0.23, p=0.015	r=-0.13, p=0.015	r=-0.07, p=0.281	r=-0.15, p=0.069	r=-0.04, p=0.382
	HCP	**r=-0.36, p<0.001**	**r=-0.30, p<0.001**	**r=-0.40, p=0.001**	r=-0.20, p=0.035	r=-0.26, p=0.007	r=-0.15, p=0.051	r=-0.28, p=0.017
Input (Extended functional model)	MICS	**r=-0.45, p<0.001**	**r=-0.42, p<0.001**	**r=-0.54, p<0.001**	**r=-0.22, p<0.001**	**r=-0.41, p<0.001**	r=-0.23, p=0.086	r=-0.23, p=0.061
	HCP	**r=-0.39, p<0.001**	**r=-0.28, p=0.004**	**r=-0.47, p<0.001**	r=-0.29, p=0.011	r=-0.20, p=0.007	r=-0.12, p=0.310	r=-0.13, p=0.180
Output (Extended functional model)	MICS	r=-0.12, p=0.200	r=-0.18, p=0.131	r=-0.02, p=0.035	r=0.02, p=0.220	r=-0.23, p=0.725	r=0.04, p=0.058	r=0.15, p=0.857
	HCP	**r=-0.36, p<0.001**	**r=-0.32, p=0.001**	**r=-0.43, p<0.001**	r=-0.23, p=0.033	**r=-0.21, p=0.004**	r=-0.12, p=0.240	r=-0.19, p=0.061

Note: p-values reflect a two-sided comparison with 10,000 permutations. Significance (in bold) was deemed where p<0.004, which reflects a Bonferroni correction for seven two-side tests (each row of the table), with an alpha level of 0.05.

Supplementary Table 3: Imbalance of connectivity across cortical types

Measure of connectivity	Dataset	Visual	Somato-motor	Dorsal attention	Ventral attention	Limbic	Fronto-parietal	Default mode
E_{NAV} (Structural model)	MICS	KL=0.069, p=0.994	KL=0.011, p=0.464	KL=0.026, p=0.870	KL=0.006, p=0.548	KL=0.064, p=0.913	KL=0.007, p=0.749	KL=0.002, p=0.214
	HCP	KL=0.089, p=0.741	KL=0.022, p=0.282	KL=0.057, p=0.752	KL=0.016, p=0.352	KL=0.083, p=0.520	KL=0.014, p=0.442	KL=0.004, p=0.104
Input (Functional model)	MICS	KL=0.003, p=0.033	KL=0.024, p=0.660	KL=0.021, p=0.828	KL=0.032, p>0.999	KL=0.048, p>0.999	KL=0.025, p=0.477	KL=0.048, p=0.910
	HCP	KL=0.033, p=0.411	KL=0.032, p=0.809	KL=0.017, p=0.548	KL=0.092, p>0.999	KL=0.019, p=0.677	KL=0.039, p=0.822	KL=0.022, p=0.827
Output (Functional model)	MICS	KL=0.012, p=0.224	KL=0.062, p=0.987	KL=0.050, p>0.999	KL=0.106, p>0.999	KL=0.014, p=0.108	KL=0.040, p=0.761	**KL=0.003, p=0.001**
	HCP	KL=0.043, p=0.326	KL=0.131, p>0.999	KL=0.056, p=0.861	KL=0.096, p>0.999	KL=0.018, p=0.128	KL=0.031, p=0.423	**KL=0.004, p<0.001**
Input (Extended functional model)	MICS	KL=0.013, p=0.221	KL=0.017, p=0.695	KL=0.004, p=0.999	KL=0.064, p>0.999	KL=0.040, p=0.924	KL=0.029, p>0.999	KL=0.013, p=0.841
	HCP	KL=0.111, p=0.869	KL=0.092, p=0.695	KL=0.116, p=0.978	KL=0.194, p>0.999	KL=0.036, p=0.052	KL=0.091, p=0.834	KL=0.045, p=0.001
Output (Extended functional model)	MICS	KL=0.040, p=0.513	KL=0.051, p=0.887	KL=0.085, p>0.999	KL=0.108, p>0.999	KL=0.022, p=0.209	KL=0.061, p>0.999	**KL=0.008, p<0.001**
	HCP	KL=0.056, p=0.337	KL=0.158, p>0.999	KL=0.117, p=0.978	KL=0.150, p>0.999	KL=0.029, p=0.078	KL=0.073, p=0.612	**KL=0.032, p<0.001**

Note: p-values reflect a one-sided comparison with 10,000 permutations. Significance (in bold) was deemed where p<0.007, which reflects a Bonferroni correction for seven one-side tests (tests within a row of the table), with an alpha level of 0.05.
