# Supplementary material for: The architecture of the human default mode network explored through cytoarchitecture, wiring and signal flow
Source: Nat Neurosci. 2025 Jan 28;28(3):654–64. doi: 10.1038/s41593-024-01868-0 (PMC11893468; doi:10.1038/s41593-024-01868-0)
Supplement: Supplementary file 2 — Reporting Summary [file 41593_2024_1868_MOESM2_ESM.pdf]

nature portfolio

Corresponding author(s): Casey Paquola

Last updated by author(s): Nov 4, 2024

Reporting Summary

Nature Portfolio wishes to improve the reproducibility of the work that we publish. This form provides structure for consistency and transparency in reporting. For further information on Nature Portfolio policies, see our Editorial Policies and the Editorial Policy Checklist.

Statistics

For all statistical analyses, confirm that the following items are present in the figure legend, table legend, main text, or Methods section.

n/a	Confirmed	
☐	☒	The exact sample size (*n*) for each experimental group/condition, given as a discrete number and unit of measurement
☐	☒	A statement on whether measurements were taken from distinct samples or whether the same sample was measured repeatedly
☐	☒	The statistical test(s) used AND whether they are one- or two-sided<br>*Only common tests should be described solely by name; describe more complex techniques in the Methods section.*
☐	☒	A description of all covariates tested
☐	☒	A description of any assumptions or corrections, such as tests of normality and adjustment for multiple comparisons
☐	☒	A full description of the statistical parameters including central tendency (e.g. means) or other basic estimates (e.g. regression coefficient) AND variation (e.g. standard deviation) or associated estimates of uncertainty (e.g. confidence intervals)
☐	☒	For null hypothesis testing, the test statistic (e.g. *F*, *t*, *r*) with confidence intervals, effect sizes, degrees of freedom and *P* value noted<br>*Give P values as exact values whenever suitable.*
☒	☐	For Bayesian analysis, information on the choice of priors and Markov chain Monte Carlo settings
☒	☐	For hierarchical and complex designs, identification of the appropriate level for tests and full reporting of outcomes
☐	☒	Estimates of effect sizes (e.g. Cohen's *d*, Pearson's *r*), indicating how they were calculated

Our web collection on statistics for biologists contains articles on many of the points above.

Software and code

Policy information about availability of computer code

Data collection	No software was used to collect data
Data analysis	Custom code for data analysis was written using MATLAB2022a. All custom code is provided in an open GitHub repository (https://github.com/caseypaquola/DMN).<br>MRI data were processed using dcm2niix (v1.0.20190902), Freesurfer (v6.0), FSL (v6.0.2), AFNI (v20.3.03), MRtrix (3.0.1) and TAPAS (v6.0.1)

For manuscripts utilizing custom algorithms or software that are central to the research but not yet described in published literature, software must be made available to editors and reviewers. We strongly encourage code deposition in a community repository (e.g. GitHub). See the Nature Portfolio guidelines for submitting code & software for further information.

Data

Policy information about availability of data

All manuscripts must include a data availability statement. This statement should provide the following information, where applicable:

- Accession codes, unique identifiers, or web links for publicly available datasets
- A description of any restrictions on data availability
- For clinical datasets or third party data, please ensure that the statement adheres to our policy

All data that support the findings of this study are openly available. BigBrain is available with LORIS (https://bigbrain.loris.ca/main.php55) with preprocessed BigBrain data available in through the BigBrainWarp GitHub repository (https://github.com/caseypaquola/BigBrainWarp56). The MICS dataset is available with

CONP Portal (https://portal.conp.ca/dataset?id=projects/mica-mics130) and the HCP dataset is available with Connectome DB (https://db.humanconnectome.org/)90.

Research involving human participants, their data, or biological material

Policy information about studies with human participants or human data. See also policy information about sex, gender (identity/presentation), and sexual orientation and race, ethnicity and racism.

Reporting on sex and gender	In the manuscript, we report the proportions of each sex for each cohort. Sexes were relatively balanced in every cohort. Sex was self-reported by participants. We did not collect gender information from participants. Due to limitations of sample size, we did not perform sex-specific analyses.
Reporting on race, ethnicity, or other socially relevant groupings	Race, ethnicity or other socially relevant categorical variables were not used in the present study.
Population characteristics	Mean±SD age=30.4±6.7 years (primary dataset), 28.8±3.8 years (secondary dataset) and 28±6.3.0 (replication dataset)
Recruitment	For the present study, we recruited healthy individuals in the Montreal area via university networks. No self-selection or other recruitment biases are relevant to the present results.
Ethics oversight	The Ethics Committee of the Montreal Neurological Institute and Hospital approved the study. Written informed consent, including a statement for openly sharing all data in anonymized form, was obtained from all participants.

Note that full information on the approval of the study protocol must also be provided in the manuscript.

Field-specific reporting

Please select the one below that is the best fit for your research. If you are not sure, read the appropriate sections before making your selection.

☒ Life sciences ☐ Behavioural & social sciences ☐ Ecological, evolutionary & environmental sciences

For a reference copy of the document with all sections, see nature.com/documents/nr-reporting-summary-flat.pdf

Life sciences study design

All studies must disclose on these points even when the disclosure is negative.

Sample size	No sample size calculations were performed, because the analyses were based on comparison between modalities (rather than between individuals). As such the power of our statistical analyses was related to the density of sampling across the cortex. For each analysis, we used the maximum number of samples across the cortex, taking into account the spatial resolution of the underlying data.
Data exclusions	No participants or data were excluded from the analyses.
Replication	Primary group-level analyses were replicated at an individual-level in a separate cohort (n=8). All replication tests were successful and the full statistical outcomes are reported in the manuscript.
Randomization	No randomization was implemented in this study, as it was an observational study with no interventions.
Blinding	No blinding was implemented in this study, as it was an observational study with no interventions.

Reporting for specific materials, systems and methods

We require information from authors about some types of materials, experimental systems and methods used in many studies. Here, indicate whether each material, system or method listed is relevant to your study. If you are not sure if a list item applies to your research, read the appropriate section before selecting a response.

Materials & experimental systems

n/a	Involved in the study
☒	☐ Antibodies
☒	☐ Eukaryotic cell lines
☒	☐ Palaeontology and archaeology
☒	☐ Animals and other organisms
☒	☐ Clinical data
☒	☐ Dual use research of concern
☒	☐ Plants

Methods

n/a	Involved in the study
☒	☐ ChIP-seq
☒	☐ Flow cytometry
☐	☒ MRI-based neuroimaging

Magnetic resonance imaging

Experimental design

Design type: Structural MRI, diffusion MRI and resting-state fMRI (rsfMRI)

Design specifications: For rsfMRI, the length of scan time was 7min, 14.4min and 6min for MICs, HCP and the 7T dataset, respectively.

Behavioral performance measures: n/a

Acquisition

Imaging type(s): Structural MRI, diffusion MRI and resting-state fMRI (rsfMRI)

Field strength: 3T and 7T

Sequence & imaging parameters:

Primary MRI analyses were conducted on 40 healthy adults from the microstructure informed connectomics (MICs) cohort (14 females, mean±SD age=30.4±6.7, 2 left-handed). Scans were completed at the Brain Imaging Centre of the Montreal Neurological Institute and Hospital on a 3T Siemens Magnetom Prisma-Fit equipped with a 64-channel head coil. Two T1w scans with identical parameters were acquired with a 3D-MPRAGE sequence (0.8mm isotropic voxels, TR=2300ms, TE=3.14ms, TI=900ms, flip angle=9°, iPAT=2, matrix=320×320, 224 sagittal slices, partial Fourier=6/8). T1w scans were visually inspected to ensure minimal head motion before they were submitted to further processing. A spin-echo echo-planar imaging sequence with multi-band acceleration was used to obtain DWI data, consisting of three shells with b-values 300, 700, and 2000s/mm2 and 10, 40, and 90 diffusion weighting directions per shell, respectively (1.6mm isotropic voxels, TR=3500ms, TE=64.40ms, flip angle=90°, refocusing flip angle=180°, FOV=224×224 mm2, slice thickness=1.6mm, multiband factor=3, echo spacing=0.76ms, number of b0 images=3). One 7 min rs-fMRI scan was acquired using multiband accelerated 2D-BOLD echo-planar imaging (3mm isotropic voxels, TR=600ms, TE=30ms, flip angle=52°, FOV=240×240mm2, slice thickness=3mm, multiband factor=6, echo spacing=0.54ms). Participants were instructed to keep their eyes open, look at a fixation cross, and not fall asleep. Two spin-echo images with reverse phase encoding were also acquired for distortion correction of the rs-fMRI scans (phase encoding=AP/PA, 3mm isotropic voxels, FOV=240×240mm2, slice thickness=3mm, TR=4029 ms, TE=48ms, flip angle=90°, echo spacing=0.54 ms, bandwidth= 2084 Hz/Px).

Secondary MRI analyses were conducted in 100 unrelated healthy adults (66 females, mean±SD age=28.8±3.8 years) from the minimally preprocessed S900 release of the Human Connectome Project (HCP) . MRI data were acquired on the HCP's custom 3T Siemens Skyra equipped with a 32-channel head coil. Two T1w images with identical parameters were acquired using a 3D-MPRAGE sequence (0.7mm isotropic voxels, TE=2.14ms, TI=1000ms, flip angle=8°, iPAT=2, matrix=320×320, 256 sagittal slices; TR=2400ms,). Two T2w images were acquired using a 3D T2-SPACE sequence with identical geometry (TR=3200ms, TE=565ms, variable flip angle, iPAT=2). A spin-echo EPI sequence was used to obtain diffusion weighted images, consisting of three shells with b-values 1000, 2000, and 3000s/mm2 and up to 90 diffusion weighting directions per shell (TR=5520ms, TE=89.5ms, flip angle=78°, refocusing flip angle=160°, FOV=210×180, matrix=178×144, slice thickness=1.25mm, mb factor=3, echo spacing=0.78ms). Four rs-fMRI scans were acquired using multi-band accelerated 2D-BOLD echo-planar imaging (2mm isotropic voxels, TR=720ms, TE=33ms, flip angle=52°, matrix=104×90, 72 sagittal slices, multiband factor=8, 1200 volumes/scan, 3456 seconds). Only the first session was investigated in the present study. Participants were instructed to keep their eyes open, look at a fixation cross, and not fall asleep. Nevertheless, some subjects were drowsy and may have fallen asleep121, and the group-averages investigated in the present study do not address these inter-individual differences.

Individual-level replication analyses were conducted on 8 healthy adults (5 females, mean±SD age=28±6.3, 1 left-handed). Scans were completed at the Brain Imaging Centre of the Montreal Neurological Institute and Hospital on a 7T Siemens Magnetom Terra System equipped with a 32/8 channel receive/transmit head coil. Two qT1 scans were acquired across two scanning sessions with identical 3D-MP2RAGE sequences (0.5mm isotropic voxels, TR=5170ms, TE=2.44ms, T11/2=1000/3200ms, flip angles=4°, matrix=488×488, slice thickness=0.5mm, partial Fourier=0.75). qT1 maps from the second session were linearly registered to the qT1 maps from the first session, then averaged, to enhanced the signal to noise ratio. A spin-echo echo-planar imaging sequence with multi-band acceleration was used to obtain DWI data, consisting of three shells with b-values 300, 700, and 2000s/mm2 and 10, 40, and 90 diffusion weighting directions per shell, respectively (1.1mm isotropic voxels, TR=7383ms, TE=70.6ms, flip angle=90°, matrix=192×192, slice thickness=1.1mm, multiband factor=2, echo spacing=0.26ms, number of b0 images=3, partial Fourier=0.75). One 6 min rs-fMRI scan was acquired using multi-echo, multiband accelerated 2D-BOLD echo-planar

imaging (1.9mm isotropic voxels, TR=1690ms, TE1/2/3=10.8/27.3/43.8ms, flip angle=67°, matrix=118x118, multiband factor=3, echo spacing=0.54ms, partial Fourier=0.75). Participants were instructed to keep their eyes open, look at a fixation cross, and not fall asleep. Two multiband accelerated spin-echo images with reverse phase encoding were also acquired for distortion correction of the rs-fMRI scans.

Area of acquisition: Whole brain

Diffusion MRI: ☒ Used ☐ Not used

Parameters: MICs: b-values 300, 700, and 2000s/mm2 and 10, 40, and 90 diffusion weighting directions per shell
HCP: three shells with b-values 1000, 2000, and 3000s/mm2 and up to 90 diffusion weighting directions per shell
7T: three shells with b-values 1000, 2000, and 3000s/mm2 and up to 90 diffusion weighting directions per shell

Preprocessing

Preprocessing software: Preprocessing was conducted with micapipe for the MICs and 7T data and with the HCP minimally-processed pipeline for the HCP dataset. These packages furthermore depend on FreeSurfer, ANTs and FSL.

Normalization: Nonlinear transformation matrices were generated between DWI space and native T1w space to align tissue type segmentations and parcellations to DWI images.
Nonlinear surface registrations were used to align structural MRI and rsfMRI to a standard template.

Normalization template: We used fsaverage as a standard surface template.

Noise and artifact removal: For 3T datasets, rsfMRI timeseries were corrected for gradient nonlinearity, head motion, bias field and scanner drifts, then structured noise components were removed using ICA-FIX, further reducing the influence of motion, non-neuronal physiology, scanner artefacts and other nuisance sources. A similar procedure was used for the 7T dataset, however, the "tedana" software package was used rather than ICA-FIX, because tedana it is optimised for multi-echo data.

All DWI scans underwent b0 intensity normalization and were corrected for susceptibility distortion, head motion, and eddy currents.

Volume censoring: The first five volumes of each rsfMRI scan were discarded to ensure magnetic field saturation. No further volume censoring was employed.

Statistical modeling & inference

Model type and settings: Product-moment correlations were performed between cortical maps.

Effect(s) tested: The strength of correlation between cortical maps.

Specify type of analysis: ☐ Whole brain ☐ ROI-based ☒ Both

Anatomical location(s): Whole brain as well as analyses focused on the default mode network

Statistic type for inference (See Eklund et al. 2016): Significance thresholds were set at $p<0.05$ for one-sided tests and $p<0.025$ for two-sided tests.

Correction: Spin permutation testing was used to correct the statistical analyses, whereby the correction accounts for the known spatial autocorrelation of cortical maps.

Models & analysis

n/a	Involved in the study
☐	☒ Functional and/or effective connectivity
☒	☐ Graph analysis
☒	☐ Multivariate modeling or predictive analysis

Functional and/or effective connectivity: For functional connectivity analyses, product-moment correlations were used.
For effective connectivity analyses, regression dynamic causal modelling was used.
